# Supplementary material for: Key factors influencing the prescribing of statins: a qualitative study among physicians working in primary healthcare facilities in Indonesia
Source: BMJ Open. 2020 Jun 15;10(6):e035098. doi: 10.1136/bmjopen-2019-035098 (PMC7299032; doi:10.1136/bmjopen-2019-035098)
Supplement: Author's manuscript [file bmjopen-2019-035098.draft_revisions.pdf]

BMJ Open

BMJ Open is committed to open peer review. As part of this commitment we make the peer review history of every article we publish publicly available.

When an article is published we post the peer reviewers' comments and the authors' responses online. We also post the versions of the paper that were used during peer review. These are the versions that the peer review comments apply to.

The versions of the paper that follow are the versions that were submitted during the peer review process. They are not the versions of record or the final published versions. They should not be cited or distributed as the published version of this manuscript.

BMJ Open is an open access journal and the full, final, typeset and author-corrected version of record of the manuscript is available on our site with no access controls, subscription charges or pay-per-view fees (http://bmjopen.bmj.com).

If you have any questions on BMJ Open's open peer review process please email info.bmjopen@bmj.com

BMJ Open

Key factors influencing the prescribing of statins: a qualitative study among physicians working in primary health care facilities in Indonesia

Journal:	*BMJ Open*
Manuscript ID	bmjopen-2019-035098
Article Type:	Original research
Date Submitted by the Author:	18-Oct-2019

Complete List of Authors:	Irawati, Sylvi; University of Groningen,<br>Prayudeni, Sari; Universitas Surabaya Kampus UBAYA Tenggilis<br>Rachmawati, Riris; Optima Pharmacy<br>Wita, I ; Udayana University Faculty of Medicine<br>Willfert, Bob; University of Groningen<br>Hak, Eelko; Rijksuniversiteit Groningen, Faculty of Mathematics and Natural Sciences<br>Taxis, Katja; University of Groningen, Faculty of Mathematics and Natural Sciences

Keywords:	Ischaemic heart disease < CARDIOLOGY, Health policy < HEALTH SERVICES ADMINISTRATION & MANAGEMENT, Protocols & guidelines < HEALTH SERVICES ADMINISTRATION & MANAGEMENT, Quality in health care < HEALTH SERVICES ADMINISTRATION & MANAGEMENT, QUALITATIVE RESEARCH

SCHOLARONE™
Manuscripts

I, the Submitting Author has the right to grant and does grant on behalf of all authors of the Work (as defined in the below author licence), an exclusive licence and/or a non-exclusive licence for contributions from authors who are: i) UK Crown employees; ii) where BMJ has agreed a CC-BY licence shall apply, and/or iii) in accordance with the terms applicable for US Federal Government officers or employees acting as part of their official duties; on a worldwide, perpetual, irrevocable, royalty-free basis to BMJ Publishing Group Ltd ("BMJ") its licensees and where the relevant Journal is co-owned by BMJ to the co-owners of the Journal, to publish the Work in this journal and any other BMJ products and to exploit all rights, as set out in our licence.

The Submitting Author accepts and understands that any supply made under these terms is made by BMJ to the Submitting Author unless you are acting as an employee on behalf of your employer or a postgraduate student of an affiliated institution which is paying any applicable article publishing charge ("APC") for Open Access articles. Where the Submitting Author wishes to make the Work available on an Open Access basis (and intends to pay the relevant APC), the terms of reuse of such Open Access shall be governed by a Creative Commons licence – details of these licences and which Creative Commons licence will apply to this Work are set out in our licence referred to above.

Other than as permitted in any relevant BMJ Author's Self Archiving Policies, I confirm this Work has not been accepted for publication elsewhere, is not being considered for publication elsewhere and does not duplicate material already published. I confirm all authors consent to publication of this Work and authorise the granting of this licence.

For peer review only

Key factors influencing the prescribing of statins: a qualitative study among physicians working in primary health care facilities in Indonesia

Sylvi Irawati,[1,2,3*] Sari Prayudeni,[4*] Riris Rachmawati,[5] I Wayan Wita,[6] Bob Wilffert,[3,7] Eelko Hak,[3] Katja Taxis[3]

*shared authorship

[1]Universitas Surabaya, Centre for Medicines Information and Pharmaceutical Care, Faculty of Pharmacy, Surabaya, Indonesia

[2]Universitas Surabaya, Department of Clinical and Community Pharmacy, Faculty of Pharmacy, Surabaya, Indonesia

[3]University of Groningen, Groningen Research Institute of Pharmacy, PharmacoTherapy, -Epidemiology & -Economics, Groningen, the Netherlands

[4]Universitas Surabaya, Faculty of Pharmacy, Surabaya, Indonesia

[5]Optima Pharmacy, Surabaya, Indonesia

[6]Udayana University, Department of Cardiovascular Medicine, Denpasar, Indonesia

[7]University of Groningen, Department of Clinical Pharmacy & Pharmacology, University Medical Center Groningen, Groningen, The Netherlands

Corresponding author:

Sylvi Irawati

Email: s.irawati@rug.nl

Phone: +31 638649411

ORCID ID: 0000-0001-6278-9017

For peer review only - http://bmjopen.bmj.com/site/about/guidelines.xhtml

ABSTRACT

Objectives: To elicit key factors influencing physicians' decision to prescribe statins.

Design: A qualitative study using a phenomenological approach within a pragmatism interpretive framework. A combination of purposive and snowball sampling was used to recruit physicians. Data were collected through face-to-face, semi-structured interviews with physicians working in primary health care facilities in a capital of a province in Indonesia. We recorded and verbatim transcribed the interviews. Coding was done independently by two researchers and data were analyzed using phenomenological data analyses. Key factors influencing physicians' decision to prescribe statins were classified into factors at the micro, meso, and macro levels according to the structural model by Scoggins et al.

Participants and setting: Physicians working in primary health care facilities in a capital of a province in Indonesia.

Results: Ten physicians were included in the study. Key factors at the micro level were that physicians knew guidelines in general, but there was uncertainty how to take into account the level of total cholesterol in combination with other cardiovascular risk factors such as diabetes and hypertension. At the macro level, the new National Health Insurance system (NHIS) appeared to facilitate the prescription of statins though more clinical information should be integrated in the system's platform to support appropriate prescribing.

Conclusions: The findings indicate lack of awareness of specific details in current guideline recommendations. Appropriate prescribing of statins should be enhanced using the new NHIS.

Keywords: cardiovascular diseases, physicians, drug prescribing, statins, qualitative research

For peer review only - http://bmjopen.bmj.com/site/about/guidelines.xhtml

Strengths and limitations of this study

- This is the first study to gain insights from physicians on key factors influencing their decision in prescribing statins in a LMIC setting.
- Face-to-face interviews by an interviewer who was known to physicians made it possible to gain more trustworthy information.
- Follow-up interviews, member checking process, independent coders, discussion with several researchers to reach consensus and the use of a theoretical framework improved the credibility of the results.
- Despite code saturation was appropriately reached with a small sample size, a larger sample size is needed to obtain meaning saturation to provide a deeper understanding of the investigated phenomenon.

For peer review only

INTRODUCTION

Despite the fact that the use of statins has been endorsed by local and various international guidelines[1–6] due to their effectiveness in preventing the first or a recurrent CVD event or CVD death,[7–9] many studies demonstrate either under- or over-prescribing of this drug class, especially in groups with moderate-to-very high risk of CVD in the European general population and in an outpatient setting in the United States.[10–13] One small study (N = 243 cases) with medical records data taken from three private hospitals in Jakarta, the capital city of the Republic of Indonesia, a lower-middle-income country (LMIC), suggested patients might not be properly treated with statins. But under- or over-prescribing was not clearly defined, because data required to determine the appropriateness to prescribe statins are not routinely measured or well documented.[14 15] Another study in ambulatory diabetes patients in Surabaya revealed the phenomenon of under-prescribing with only 25% of eligible patients receiving statins.[16] Studies investigating the consequences of under- and over-prescribing of statins are scarce. Nevertheless, one case study in a United Kingdom population with a ≥ 20% of 10-y risk of developing CVD illustrates that under-prescribing will substantially reduce quality-adjusted life years (QALYs) gained over a 9 year period (2006-2014) and over-prescribing will certainly increase the cost burden of prescription between £80 million and £117 million annually without certainty whether the benefit of prevented CVD events outweighs the adverse drug reaction.[17]

Factors influencing physicians' prescribing behavior, regardless of the medication being prescribed, has been investigated since the 1950s. Physicians' personal and professional characteristics, pharmaceutical marketing strategies (direct-to-physician or direct-to-consumer marketing), drug information sources, drug characteristics, drug price, and patients' characteristics have been identified as factors influencing physicians' prescribing behavior, although there is inconsistency over the relative importance of each factor.[18–24] More importantly, the majority of studies in this field have been conducted in high-income Western countries with a different culture, health care and health insurance systems compared to LMICs.

In order to reach universal health coverage (UHC) in 2019, the Government of Indonesia launched a new mandatory National Health Insurance System (NHIS) program (*Jaminan Kesehatan Nasional-Kartu Indonesia Sehat*, *JKN-KIS*) run by the Indonesian Social Security Agency for Health (*Badan Pengelola Jaminan Sosial*, *BPJS Kesehatan*) in 2014.[25–27] The system facilitates more patients to

For peer review only

go to the primary health care facilities first instead of directly visiting specialists in the secondary facilities.[25 28] Moreover, more medicines, listed in the 2014 revised edition of the National Formulary, are insured by the government to improve access to medications for patients.[25 29 30] Based on the National Formulary, two types of statins are insured (pravastatin and simvastatin). A total cardiovascular (CV) risk score is not mentioned to guide statin prescribing.[29 30] This policy is different compared to the new clinical guideline for dyslipidemic patients issued by the Indonesian Heart Association (IHA) which adapted guidelines issued by the European Society of Cardiology (ESC) and the European Atherosclerosis Society (EAS).[2 31] In this guideline, statins should be prescribed according to both the level of LDL-C and the total CV risk score. Thus, this situation may create confusion for physicians in prescribing statins and increase the risk of under- or over-prescribing of statins. In the current qualitative study, we aimed to understand the key factors influencing physicians to prescribe statins, especially in the primary health care facilities in a LMIC.

ETHICS APPROVAL

This study was approved by the local Indonesian Medical Association (IMA). The aim of the study was known to all participants before the interview was started. Informed consents were obtained from each participants. Participant's personal identities were kept anonymous and all transcripts were stored and maintained confidential.

For peer review only - http://bmjopen.bmj.com/site/about/guidelines.xhtml

METHODS

Study design and framework

This was a qualitative study using a phenomenological approach within a pragmatism interpretive framework. The pragmatism framework focuses on the phenomenon being investigated (key factors influencing physicians in prescribing statins) and the question inquired regarding this phenomenon. This framework allowed choosing the method that worked best in the field to answer the research question.[32] We used the Standards for Reporting Qualitative Research (SRQR) checklist when writing our report.[33]

Study context and setting

As briefly introduced earlier, the Government of Indonesia set a new NHIS conducted by *BPJS Kesehatan*, namely *JKN-KIS*, in 2014. In this system, Indonesia's Ministry of Health also enforced the already existing platforms such as the National Formulary and the e-Catalogue to support cost-effective medicine and medical supply procurement throughout the country. The National Formulary lists medicines reimbursed by BPJS. The e-Catalogue is a national electronic platform required to be used by healthcare facilities for procuring medicines. The e-Catalogue lists medicines with their prices and selected pharmaceutical companies as supplier. Unless the medicines which are needed are not listed in the e-Catalogue, it is mandatory for all health care facilities to purchase medicines from this platform.[25 26]

Indonesia's primary health care is delivered by public and private primary health care facilities. Public primary health care facilities consist of primary health centers (PHCs or *puskesmas*) and their supporting networks (*pustu*, *puskel*, *polindes*, *poskesdes*) owned and organized by District or City Health Offices. Private primary health care facilities consist of individual practice general practitioners (GPs), midwives, and nurses; and private clinics. As a part of new NHIS program in 2014, the national referral system to access these facilities was also established. Figure 1 presents an overall picture of the health care delivery system in Indonesia.[34]

Participants

We viewed the topic of our study was sensitive in nature.[35 36] Prescribing activity in Indonesia is an authority of registered physicians governed by regulations[37 38] and ethical codes.[39 40] There might be issues which would not be frankly expressed due to its sensitivity. Thus, we selected a city, a capital of a

For peer review only - http://bmjopen.bmj.com/site/about/guidelines.xhtml

province in Indonesia, where our study had been welcomed and approved by the local physician associations of the city. We gathered information from physicians working at primary care health facilities in this city. The province was quite small with an area around 6000 ha. The city is the most populous city in the province with around 900,000 inhabitants in 2017, about 21.5% of the province's population.

We obtained the list of physicians' names registered as member of the physician associations of the city, but up-to-date physicians' personal contact details were not available. Therefore, we purposively selected physicians who worked at the primary health care facilities in the capital city who were known to a member of the research team (SP), and consented to participate in the study. Participating physicians were then asked for contact details of potential other participants (snowball sampling).

Data collection

Baseline characteristics of the participants were collected by interview (age, practice experience, educational period). Initially, an interview guide was developed by SI and SP based on general key factors in prescribing found in the literature (see Table 1).[18] A semi-structured face-to-face interview was conducted with each participant by SP between July 2015 and January 2016. To obtain the key factors considered by physicians in prescribing statins, SP only asked the main question at first. The probing questions were asked after physicians answered the first question in an explanatory way. All interviews were audio-recorded using a tape recorder. The interviewer also took some field notes. The result from the initial interview of each participant was pre-analysed directly by SI and SP to confirm whether it had covered the aim of the study and to determine if a follow-up interview with the same participant was needed. Data collection was discontinued when code saturation was reached as described by Hennink et al.[41]

Data analysis

The recorded interviews were listened to repeatedly and transcribed verbatim whenever possible. The field notes were checked to add information to the transcripts. To enhance trustworthiness and credibility of data analysis, a technique known as member checking was applied by returning each transcript to the respective participant to confirm the content of the interview.

For peer review only - http://bmjopen.bmj.com/site/about/guidelines.xhtml

Using the phenomenological approach, we highlighted and coded keywords which indicated key factors in prescribing statins from the transcripts. The same code was assigned to the same keyword. We categorized the codes in minor subthemes, subthemes and final major themes. The encoding process until obtaining subthemes was conducted independently by SP and RR. Disagreement regarding the codes and subthemes was discussed between SI and SP to reach a consensus. After subthemes were drawn, SP, SI, and IWW discussed major themes and compared the themes with the findings from similar qualitative studies.

The final themes were refined by SI and KT and were mapped into a model that had previously been developed by Scoggins et al.[42] This was a qualitative study, investigating factors influencing GPs to prescribe statins in England using in-depth interviews and focus group discussions. Interviews were done with senior managers primary care service organisations (Primary Care Trusts, PCTs), focus group discussions were done with GPs of those organisations. The factors were classified into macro, meso and micro level (Figure 2).

RESULTS

Ten physicians (nine females, one male) were willing to participate in this study. The age of seven physicians was ≤ 30 years old, two were between 31 and 40, and one between 51 and 60 years old. Five physicians graduated between 2010 and 2015, four between 2000-2010, and one between 1980 and 1990.

Table 2 presents all major themes, subthemes, and minor subthemes derived from transcript analysis. In general, there were two major themes drawn from the analysis: factors operating at (a) micro and (b) macro levels. We did not identify any themes on the meso level. Figure 2 shows the themes mapped into the model by Scoggins et al.[42]

Theme 1: Key factors in statin prescribing at the micro level

The physicians described the key factor influencing their decision to prescribe statins as their inherent understanding of the characteristics of patients which indicate the patient's need for a statin or a contra-indication. There was some uncertainty around these characteristics which was inseparable from other factors like motivation and education. See for further explanation below.

For peer review only - http://bmjopen.bmj.com/site/about/guidelines.xhtml

Subtheme: Patient's characteristics

Physicians described several patient's characteristics which they considered to be important for prescribing statins. The most important characteristic was a high level of total cholesterol (TC), other characteristics were clinical symptoms, comorbidities, and other risk factors.

> "From the level of cholesterol of the patient him/herself, if for example, it is more than 200 [mg/dL], for example 210, but it looks like it still can be [controlled] with dietary pattern, I have not prescribed it yet. However, if, for example, there have been symptoms such as [on the] neck and maybe he/she has high blood pressure, usually a medication [is prescribed] – simvastatin 1 x 10 mg. Actually, from my personal perspective, I do not look at the high blood pressure only. Only from the level of cholesterol, if, for example, it has been already high such as up to 250, 260, I give [the medication] even though the blood pressure is not high." (Physician 1)

Interestingly, there is disagreement over the cut-off of the TC level to initiate statins. Other disagreements were also found on how these factors related to each other when influencing the decision to prescribe statins. These variations of considerations were captured in the following quotes:

> "It depends on the patient whether there is a comorbidity or not. If there is a comorbidity, usually [with the level of cholesterol] above 200, [statin] has already been prescribed. If not, maybe above 240. If there is hypertension, diabetes, above 200 also [it will] be directly given." (Physician 8)

> "If, for example, the tryglycerides level is above 150, it can be prescribed. Then, after that... tryglycerides and total cholesterol. [For total cholesterol] usually above 200, it is prescribed." (Physician 10)

In prescribing statins, a physician also considered a situation where statin administration was contraindicated as quoted below:

For peer review only

"First, there has to be a clear or an appropriate indication. Then, whether there is a contraindication for the patient to use the medication for example in patients with liver disorder, then in patients who are pregnant – this is also a relative contraindication for statin administration." (Physician 6)

Subtheme: Professional experience of the prescribing physicians

This subtheme focused more on the personal choice of physicians. A mindset of cardiovascular event prevention motivated physicians to prescribe statins as described below:

"I give statins to the patient with high cholesterol lipid not just to decrease the level of the cholesterol. I mean that, you know that patient with high cholesterol lipid has much more risk for cardiovascular incident, because, in my knowledge, if the cholesterol level in the blood is so high, [it] can cause the plaque in the vascular, in the blood vessels. If the plaque becomes unstable, [it] can cause a rupture and make a mismatched perfusion and demand. So, I give the statin for the cholesterolemias, uh... the high cholesterol level in the laboratory test, other reason – no other reason, I think. It's the main reason. It's my first reason to give [is] to prevent. It prevents the cardio, the risk of cardiovascular incident." (Physician 4)

"... for statin administration as a medication to manage dyslipidemia, especially in patients with dyslipidemia or in patients with high risk of cardiovascular disease events that it can be as preventive therapy in cardiovascular disease, such as in patients who have already had a coronary heart disease or in patients with diabetes mellitus and… and with a risk, with peripheral artery disease so to say, hence there is a place to give [statin] or in patients with stroke." (Physician 6)

Theme 2: Key factors in statin prescribing operating at the macro level

We found that international information or references framed the physician's mindset of patients who were candidates to receive statins. Furthermore, the NHIS supported or hindered the decision to prescribe statins.

For peer review only - http://bmjopen.bmj.com/site/about/guidelines.xhtml

Subtheme: References

References including journals and guidelines were information sources which may frame physicians' knowledge on which patients to prescribe statins for. These factors were not mentioned initially by participants but elicited through probing questions. The sources mentioned included therapeutic guidelines covered during medical education as well as local and global standards.

> "I do… as in the standard operating procedure, i.e. giving a healthy lifestyle first… a healthy dietary lifestyle. The standard operating procedure is from sources for example from journals… yes…also with medical references in the past… got from the college and when I was in the hospital [during internship as a junior physician]" (Physician 2)

> "In the past Framingham score was used to determine the risk for cardiovascular events. Now there is a new published guideline where [the risk] was not calculated from the Framingham score but [also] saw other risk factors. If not mistaken, [the guideline] was issued by American Heart Association also." (Physician 6)

> "There is a standard for that. If, for example, [the level of cholesterol] is above 250, the medication is just started. If it is between 200-250, counseling related to dietary pattern and lifestyle only." (Physician 9)

Subtheme: National Health Insurance System

Although the new NHIS means more patients get free access to medicines, there are still criteria to prescribe statins for reimbursement in the National Formulary which may or may not facilitate prescribing. For example, in the National Formulary it was stated that pravastatin was to be given only to hyperlipidemic patients who had LDL-c level > 160 mg/dL, to patients with coronary heart disease, and patients with diabetes and macroalbuminuria.[29 30] This suggested that physicians would tend to prescribe to this type of patients or would prescribe it anyway if the patient did not meet this strict criteria but was considered to need a statin with the consequence it would not be reimbursed by NIHS.

For peer review only - http://bmjopen.bmj.com/site/about/guidelines.xhtml

"The price is cheap, [it] is easy to obtain... in terms of it is available everywhere, then also available in generic. This also does not make it difficult for some patients even more when retired elderly patients do not have money but using *BPJS*. So, I think that is my consideration." (Physician 5)

"*BPJS* will only reimburse the administration of statins for LDL above 160. Therefore, if it is under 160, patients usually buy it by themselves, so we prescribe. Meanwhile, specialists in hospitals usually prescribe but this is not reimbursed by BPJS... so we help by prescribe it again." (Physician 8)

"No, if indeed only the cholesterol is high, I think no need to refer [the patient]. No diabetes, nothing... we usually prescribe directly, unless when simvastatin is incidentally not available in *Puskesmas*... he/she [the patient] insists because he/she has a right... sometimes the medication is not available. He/she has the right... insurance right, for example he/she wants a referral, we refer. However, there he/she also does not certainly obtain [a statin] if the LDL is under 160." (Physician 8)

A theme categorized under Pandora Box

We attempted to elicit the impact of pharmaceutical companies and their marketing strategy related to statin prescribing, but physicians preferred to not discuss this topic with the researcher.

DISCUSSION

Physicians' decision to prescribe statins was influenced by factors operating at the micro and macro levels which mapped well on the framework developed by Scoggins et al (2006) in a study on the factors influencing GPs prescribing of statins in primary care in England. The key micro level factor was a set of patient's characteristics, especially the high level of TC. At the macro level, physicians explained that their knowledge on prescribing was grounded in their education at university as well as reading guidelines or other reference sources such as medical journals. Another macro level factor was the new NHIS which facilitated or limited the prescribing of statins.

For peer review only - http://bmjopen.bmj.com/site/about/guidelines.xhtml

The strength of this study lies in the use of a qualitative method to explore processes behind the behavior of prescribing statins in Indonesia, a LMIC in transition to achieve UHC. The validity of the data was improved by follow-up interviews, member checking process, independent coders, and discussion with several researchers to reach consensus. Although we have reached code saturation with our interviews, we only interviewed a relatively small number of physicians from one city in one province in Indonesia. Nevertheless, several of our findings are comparable with other qualitative studies. Therefore, our findings are the foundation for a quantitative study to investigate the extent to which these factors play a role influencing prescribing quality of statins in Indonesia. More qualitative work on eliciting factors influencing physician's prescribing behavior in other Asian regions and other therapeutic areas could complement our findings.

Similar to Scoggins et al. (2006), our study found the clinical need of patients as an important factor to consider prescribing statins. However, physicians in our study described the clinical needs in more detail, mainly the level of TC and other risk factors potentially increasing the risk of CVD. Interestingly, the range of cholesterol levels to start statins ranged from 200 mg/dL to 260 mg/dL and there was also a variation in taking other CVD risk factors into account. For example, one physician decided to give a statin when the TC level ≥ 210 mg/dL and the patient also had clinical symptoms and comorbidities while another physician only prescribed statins when the patient's TC level was ≥ 260 mg/dL without the presence of comorbidities. This practice was not in line with the local guideline to initiate statin therapy.[2] The patient's total CV risk calculation was only acknowledged once after probing. This variation may lead to under- or over-prescribing of statins. The hesitation and inconsistency in using risk assessment tools as a guidance to initiate statin therapy has also been revealed in another qualitative study performed among GPs in the United Kingdom (UK) which showed that GPs found it difficult to interpret the tool for Asian patients and therefore preferred to assess the risk intuitively.[43] The physicians in our study had some general background knowledge on statin prescribing which was by and large in line with the National Formulary. They did not mention the use of the national guideline issued by the Indonesian Heart Association as sources to guide statin prescribing. This raises the question whether this guideline is sufficiently promoted.

Our general findings are also in agreement with a report from the World Health Organization (WHO) on the drug use situation in Indonesia in 2011. It reported non-uniform sources of drug

For peer review only - http://bmjopen.bmj.com/site/about/guidelines.xhtml

information used by physicians; they could be old text books from medical school or the internet with little use of up-to-date sources of independent drug information. The national standard treatment guideline for PHCs was not used much by physicians.[44] Even if it was used, there was no recommendation to use statins in patients with coronary heart disease as the guideline was developed when statins had not routinely been used in clinical practice.[45] Although we were unable to gauge the role of medical representatives or pharmaceutical companies as shown in other studies [24 42] in influencing statin prescribing, the WHO report suggested medical representatives from pharmaceutical companies as one of probable sources of drug information used by most physicians.[44] As the WHO report is based on a situational analysis conducted in 2011 where the NHIS was still not integrated, it is not possible to know to what extent the role of pharmaceutical companies has changed. This question may create an ethical dilemma, therefore, a collaboration with the Ministry of Health and professional bodies and councils is needed to provide a less biased answer.

Patients' demand or request for medicines is a factor influencing physicians' prescribing behavior according to Scoggins et al. (2006). This factor was not mentioned in our interviews although there was an indication that patient's insistence may influence the physician to meet the patient's expectation, e.g. in granting a referral. The lack of involvement of the patients' voice in the physicians' decision can be caused by differences in physician-patient communication style which may be rooted in cultural differences between Southeast Asians and Westerners.[46 47] The majority of studies exploring factors influencing physicians' prescribing behavior come from Western countries where the physician-patient communication style is often based on a partnership style with the concept of equity.[20 24 42 43 48 49] Southeast Asian physicians commonly tend to communicate in a paternalistic or one-way style, which gives the physician a more dominant role towards the patient. The culture of Southeast Asian physicians in keeping a social distance supports this communication style.[46 47]

We found that the NHIS had a considerable influence on physicians' prescribing decisions, in particular on the choice of generics. This is in line with the concept from Scoggins et al. (2006) that prescribing decisions are influenced by factors operating at national context or macro level. However, Scoggins et al. (2006) only list national and international evidence based on authoritative journals, national guidelines, national media, and the development of new treatments as factors. This difference

For peer review only - http://bmjopen.bmj.com/site/about/guidelines.xhtml

may be explained because of differences in organizing health care and reimbursement of medication between Indonesian and England.

In contrast to Scoggins et al.,[42] we did not identify any factors at the meso level such as benchmarking between different primary care practices, collaborations between primary care and specialists and regional formularies. This is understandable as strengthening the primary care sector is a recently developed strategy with the new NHIS in Indonesia. Therefore, activities which are common practice in the English primary care system at the meso level, have not been established in Indonesia yet. This finding is an opportunity for the government to work on the meso level to strengthen rational prescribing behaviour.

The findings from our study indicate that physicians have a lack of awareness of specific guideline recommendations, especially on the use of LDL-C level and total CV risk calculation as decisive factors to prescribe statins. Improved access to statins facilitated by the government through the new NHIS seemed to be a good first step in fostering rational prescribing. A next step would be to include the recommendations from clinical guidelines issued by IHA into this system, especially through the National Formulary.

For peer review only

For peer review only - http://bmjopen.bmj.com/site/about/guidelines.xhtml

ACKNOWLEDGEMENTS

The authors would like to thank to all physicians participated in this study.

CONTRIBUTORS

SI, SP, and IWW conceived and designed the study. SP carried out the interviews. SI, SP, RR, EH and KT analysed and interpreted the data. SI and SP drafted the manuscript. IWW, BW, EH, and KT gave critical inputs to the intellectual content in the drafted manuscript. All authors read, revised, and approved the final manuscript. SP had full access to all of the data in the study. All authors can take responsibility for the integrity of the work.

FUNDING

This research received no specific grant from any funding agency in the public, commercial, or not-for-profit sectors. SI received support from the Indonesia Endowment Fund for Education (Lembaga Pengelola Dana Pendidikan, LPDP), the Ministry of Finance of Republic of Indonesia, in the form of scholarship for her PhD program. The LPDP had no role in research design, data collection, analysis and interpretation, or manuscript writing and publication.

COMPETING INTERESTS

None declared

For peer review only

REFERENCES

1. World Health Organization. Prevention of cardiovascular disease: guidelines for assessment and management of cardiovascular risk. Geneva: WHO Press; 2007.
2. Erwinanto, Santoso A, Putranto JNE, et al. Pedoman tatalaksana dislipidemia. J Kardiol Indones [Internet]. 2013;34(4):245–70. Available from: http://jki.or.id.
3. JBS3 Board. Joint British Societies' consensus recommendations for the prevention of cardiovascular disease (JBS3). Heart. 2014;100:ii1–67.
4. Piepoli MF, Hoes AW, Agewall S, et al. 2016 European guidelines on cardiovascular disease prevention in clinical practice. Eur Heart J. 2016;37(29):2315–81.
5. Stone NJ, Robinson J, Lichtenstein AH, et al. 2013 ACC/AHA guideline on the treatment of blood cholesterol to reduce atherosclerotic cardiovascular risk in adults: a report of the American College of Cardiology/American Heart Association Task Force on Practice Guidelines. Circulation. 2014;129::S1–S45.
6. Catapano AL, Graham I, De Backer G, et al. 2016 ESC/EAS guidelines for the management of dyslipidaemias. Eur Heart J. 2016;37(39):2999–3058.
7. Chou R, Dana T, Blazina I, et al. Statins for prevention of cardiovascular disease in adults. JAMA. 2016;316(19):2008–24.
8. Mihaylova B, Emberson J, Blackwell L, et al. The effects of lowering LDL cholesterol with statin therapy in people at low risk of vascular disease: meta-analysis of individual data from 27 randomised trials. Lancet. 2012;380(9841):581–90.
9. Taylor F, Huffman M, Macedo A, et al. Statins for the primary prevention of cardiovascular disease. Cochrane Database Syst Rev. 2013;(1):1–97.
10. Tonstad S, Rosvold EO, Furu K, et al. Undertreatment and overtreatment with statins: the Oslo Health Study 2000-2001. J Intern Med. 2004;255(4):494–502.
11. Smith M, Cox E, Bartell J. Overprescribing of lipid lowering agents. Qual Saf Health Care. 2006;15(4):251–7.
12. Verma A, Visintainer P, Elarabi M, et al. Overtreatment and undertreatment of hyperlipidemia in the outpatient setting. South Med J. 2012;105(7):329–33.
13. Langsted A, Freiberg JJ, Nordestgaard BG. Extent of undertreatment and overtreatment with

For peer review only

For peer review only - http://bmjopen.bmj.com/site/about/guidelines.xhtml

cholesterol-lowering therapy according to European guidelines in 92,348 Danes without ischemic cardiovascular disease and diabetes in 2004–2014. Atherosclerosis. 2017;257:9–15.

14. Simatupang A, Irianto A, Simanjuntak W, et al. Pattern of statin use in several hospital in Jakarta: a cross sectional study. J Kedokt Yars. 2006;14(3):223–9.

15. Third report of the National Cholesterol Education Program (NCEP) expert panel on detection, evaluation, and treatment of high blood cholesterol in adults (Adult Treatment Panel III) final report. Circulation. 2002;106(25):3143–421.

16. Lie PGCE, Irawati S, Presley B. Prevention of cardiovascular disease in diabetes mellitus outpatient: focusing on antiplatelet, statins and irrational antihypertensive drug use. Indones J Clin Pharm. 2016;5(3):169–83.

17. Hodgson R, Biswas M, Morgan P. Costs and consequences of lack of adherence to prescribing guidelines for statins in the UK: a value of implementation analysis (PCV41). Value Health. 2017;20(5):A266.

18. Groves KE, MacKinnon NJ, Sketris I. Prescribing behavior. In: Rickles N, Albert I, Mickey C, editors. Social and behavioral aspects of pharmaceutical care. 2nd ed. Sudburry, USA: Jones and Bartlett Publishers; 2010. p. 141–76.

19. Hemminki E. Factors influencing drug prescribing: inquiry into research strategy. Drug Intell Clin Pharm. 1976;10(6):321–9.

20. Hemminki E. Review of literature on the factors affecting drug prescribing. Soc Sci Med. 1975;9(2):111–5.

21. Theodorou M, Tsiantou V, Pavlakis A, et al. Factors influencing prescribing behaviour of physicians in Greece and Cyprus: results from a questionnaire based survey. BMC Health Serv Res. 2009;9:150. doi: 10.1186/1472-6963-9-150.

22. Christensen DB, Bush PJ. Drug prescribing: patterns, problems and proposals. Soc Sci Med. 1981;15(3 Pt 2):343–55.

23. Schumock GT, Walton SM, Park HY, et al. Factors that influence prescribing decisions. Ann Pharmacother. 2004;38(4):557–62.

24. Murshid MA, Mohaidin Z. Models and theories of prescribing decisions: a review and suggested a new model. Pharm Pract (Granada). 2017;15(2):1–12.

For peer review only - http://bmjopen.bmj.com/site/about/guidelines.xhtml

25. Agustina R, Dartanto T, Sitompul R, et al. Universal health coverage in Indonesia: concept, progress, and challenges. Lancet. 2019;393(10166):75–102.

26. Wasir R, Irawati S, Makady A, et al. Use of medicine pricing and reimbursement policies for universal health coverage in Indonesia. PLoS One. 2019;14(2):1–19.

27. Wirtz T. The new mandatory health insurance scheme: taking stock one year after the introduction. Insurance Newsletter. PT Ernst & Young Indonesia; 2015.

28. Ekawati FM, Claramita M, Hort K, et al. Patients' experience of using primary care services in the context of Indonesian universal health coverage reforms. Asia Pac Fam Med. 2017;16(1):1–10.

29. Keputusan Menteri Kesehatan Republik Indonesia tentang Formularium Nasional. No. 328/MENKES/SK/VIII/2013. Jakarta; 2013.

30. Keputusan Menteri Kesehatan Republik Indonesia tentang perubahan atas Keputusan Menteri Kesehatan No. 328/MENKES/SK/IX/2013 tentang Formularium Nasional. No. 159/MENKES/SK/V/2014. Jakarta; 2014.

31. Reiner Ž, Catapano AL, De Backer G, et al. ESC/EAS guidelines for the management of dyslipidaemias. Eur Heart J. 2011;32(14):1769–818.

32. Creswell J, Poth CN. Qualitative inquiry and research design: choosing among five approaches. 4th ed. Los Angeles: SAGE Publication, Inc.; 2018.

33. O'Brien BC, Harris IB, Beckman TJ, et al. Standards for reporting qualitative research: a synthesis of recommendations. Acad Med. 2014;89(9):1245–51.

34. Claramita M, Syah NA, Ekawati FM, et al. Primary health care systems (Primasys): case study from Indonesia, abridged version. Geneva: World Health Organization; 2017.

35. Budianto A. Peresepan dokter: organized crime? J Huk Pembang. 2009;39(4):511–30.

36. Muh Fadhli W, Anisah S. Legal responsibilities of doctors and pharmacists in the services of recipes. Media Farm. 2016;13(1):61–87.

37. Undang-Undang Republik Indonesia tentang Praktik Kedokteran. No. 29. 2004.

38. Peraturan Menteri Kesehatan Republik Indonesia tentang Izin Praktik dan Pelaksanaan Praktik Kedokteran. No. 2052/MENKES/PER/X/2011. Jakarta; 2011.

39. Purwadianto A, Soetedjo, Gunawan S, et al. Kode etik kedokteran Indonesia. Jakarta: Pengurus

For peer review only

For peer review only - http://bmjopen.bmj.com/site/about/guidelines.xhtml

Besar Ikatan Dokter Indonesia; 2012.

40. Badan Etik dan Pembelaan Anggota Pengurus Pusat PERKI. Pedoman kode etik dokter Perhimpunan Kardiologi Indonesia (PERKI). 1st ed. Jakarta: PP. PERKI; 2014.

41. Hennink MM, Kaiser BN, Marconi VC. Code saturation versus meaning saturation: how many interviews are enough? Qual Health Res. 2017;27(4):591–608.

42. Scoggins A, Tiessen J, Ling T. Prescribing in primary care: understanding what shapes GPs' prescribing choices and how might these be changed. [Internet]. Cambridge; 2006 [cited 2019 Feb 25]. p. 1–59. Available from: https://www.rand.org/content/dam/rand/pubs/technical_reports/2007/RAND_TR443.pdf

43. Kedward J, Dakin L. A qualitative study of barriers to the use of statins and the implementation of coronary heart disease prevention in primary care. Br J Gen Pract. 2003;53(494):684–9.

44. Holloway K. Pharmaceuticals in health care delivery [Internet]. World Health Organization. 2011 [cited 2019 Sep 30]. Available from: http://searo.who.int/entity/medicines/indonesia_situational_analysis.pdf?ua=1

45. Pedoman pengobatan dasar di Puskesmas 2007. Jakarta: Departemen Kesehatan RI; 2007.

46. Claramita M, van Dalen J, van der Vleuten CP. Doctors in a Southeast Asian country communicate sub-optimally regardless of patients' educational background. Patient Educ Couns [Internet]. 2011;85(3):e169–74. Available from: http://dx.doi.org/10.1016/j.pec.2011.02.002

47. Claramita M, Nugraheni MDF, van Dalen J, et al. Doctor-patient communication in Southeast Asia: a different culture? Adv Heal Sci Educ. 2013;18(1):15–31.

48. Grant A, Sullivan F, Dowell J. An ethnographic exploration of influences on prescribing in general practice: why is there variation in prescribing practices? Implement Sci. 2013;8(1):2013.

49. AB E, Denig P, Van Vliet T, et al. Reasons of general practitioners for not prescribing lipid-lowering medication to patients with diabetes: a qualitative study. BMC Fam Pract. 2009;10:1–7.

50. Andriani H, Liao CY, Kuo HW. Association of maternal and child health center (Posyandu) availability with child weight status in Indonesia: a national study. Int J Environ Res Public Health. 2016;13(3):293. doi: 10.3390/ijerph13030293.

For peer review only - http://bmjopen.bmj.com/site/about/guidelines.xhtml

Table 1. Interview guide

Questions
Main question
• Which factors do you consider when prescribing statins to the patient ?
Probing question (not necessarily being asked, depends on answers from the main question)
• Which therapeutic guideline did you usually use in prescribing statins ?
• How do you assess a total cardiovascular risk factor ?
• Which type of statins do you often prescribe ?
• Which statin dose and duration of use you prescribe ?
• How about the relation to pharmaceutical company promotion/advertising ?
• Does the regulation in the practice setting limit the decision of therapy ?
• Have you ever found patients complaining about the cost of statins ?
• Are you having difficulties in arranging time for a consultation with the patient because there are many queues ?
• Does an insurance system such as the new national health insurance program limit you to prescribe statins to patients who need it ?

For peer review only

Table 2. Themes, subthemes and codings of factors influencing statin prescribing

Major themes	Subthemes	Minor subthemes	Specific keywords
Micro level	Patient's characteristics	Blood lipid levels	Total cholesterol (varied among participants: from 200 to 260 mg/dL; triglycerides), high LDL-c, low HDL-c, a combination of different lipid parameter
		Symptoms	Pain (varied in location: back of the neck, neck, shoulder), stiffness (neck, shoulder), warmth (neck), tingling (palms, feet), dizziness
		Comorbidities	Hypertension, diabetes, metabolic syndrome, heart disease
		Contraindication	Pregnancy, liver disease
		Other risk factors	Other risk factor without specifying the factor
	Professional experience	Primary and secondary prevention of CVD	Prevention, CVD prevention
Macro level	References	Standard	Standard
		Guideline	Guideline from ACC/AHA
		Journal	Article in journal
	National Health Insurance System	BPJS	BPJS
		e-Catalogue	e-Catalogue
		Referral system, referral back system	patient referral

ACC, American College of Cardiology; AHA, American Heart Association; BPJS, Badan Pengelola Jaminan Sosial; CVD, cardiovascular disease; HDL-c, high density lipoprotein cholesterol; LDL-c, low density lipoprotein cholesterol

For peer review only - http://bmjopen.bmj.com/site/about/guidelines.xhtml

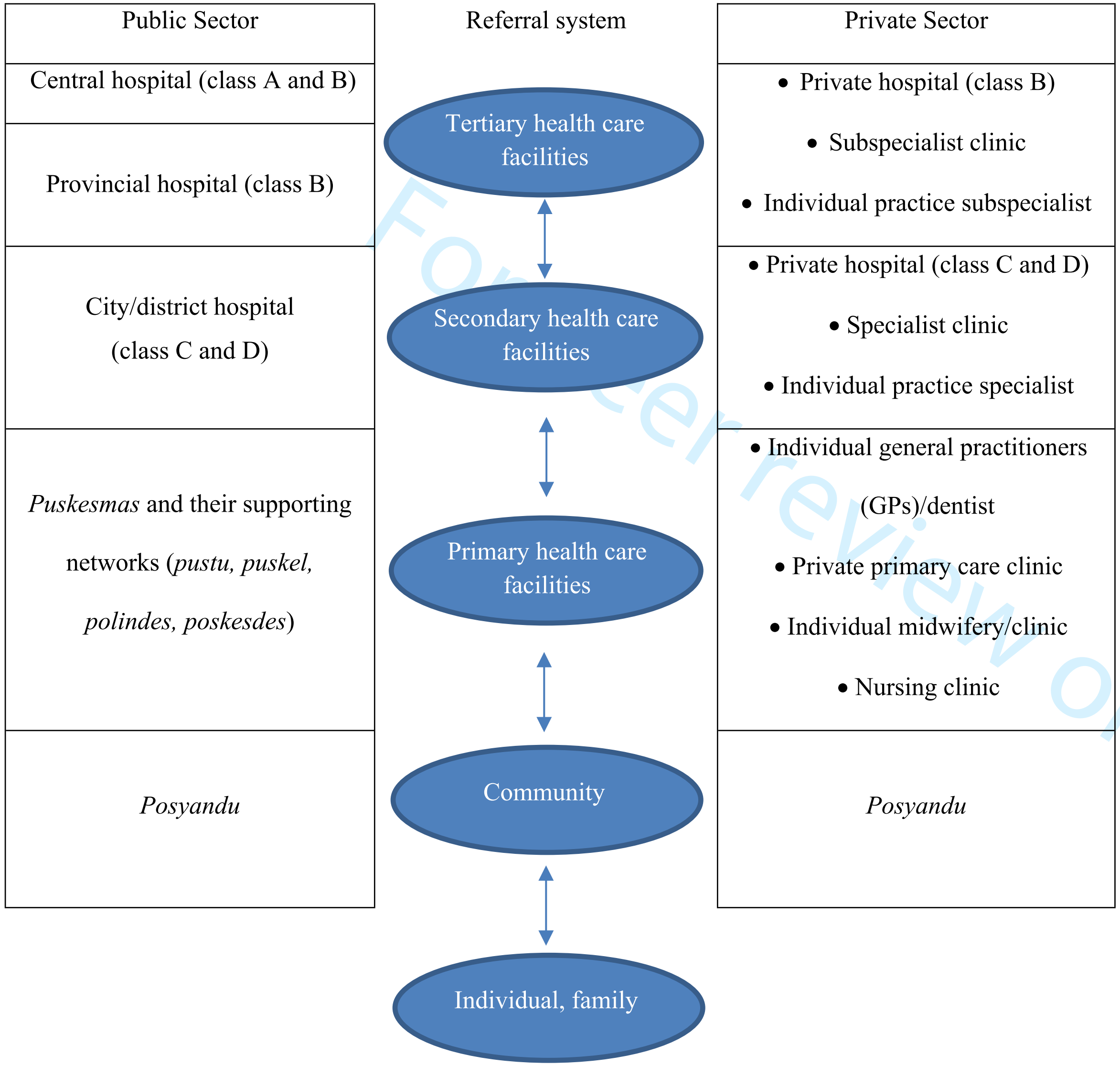

Figure 1. Health care delivery system in Indonesia, figure was modified from Claramita et al.[34]

Puskesmas is a public primary health center. *Posyandu* is a service organized by volunteers (*Kader*) to deliver health program for vulnerable groups, especially mother and children, locally. *Posyandu* is supervised by *Puskesmas*.[50]

For peer review only - http://bmjopen.bmj.com/site/about/guidelines.xhtml

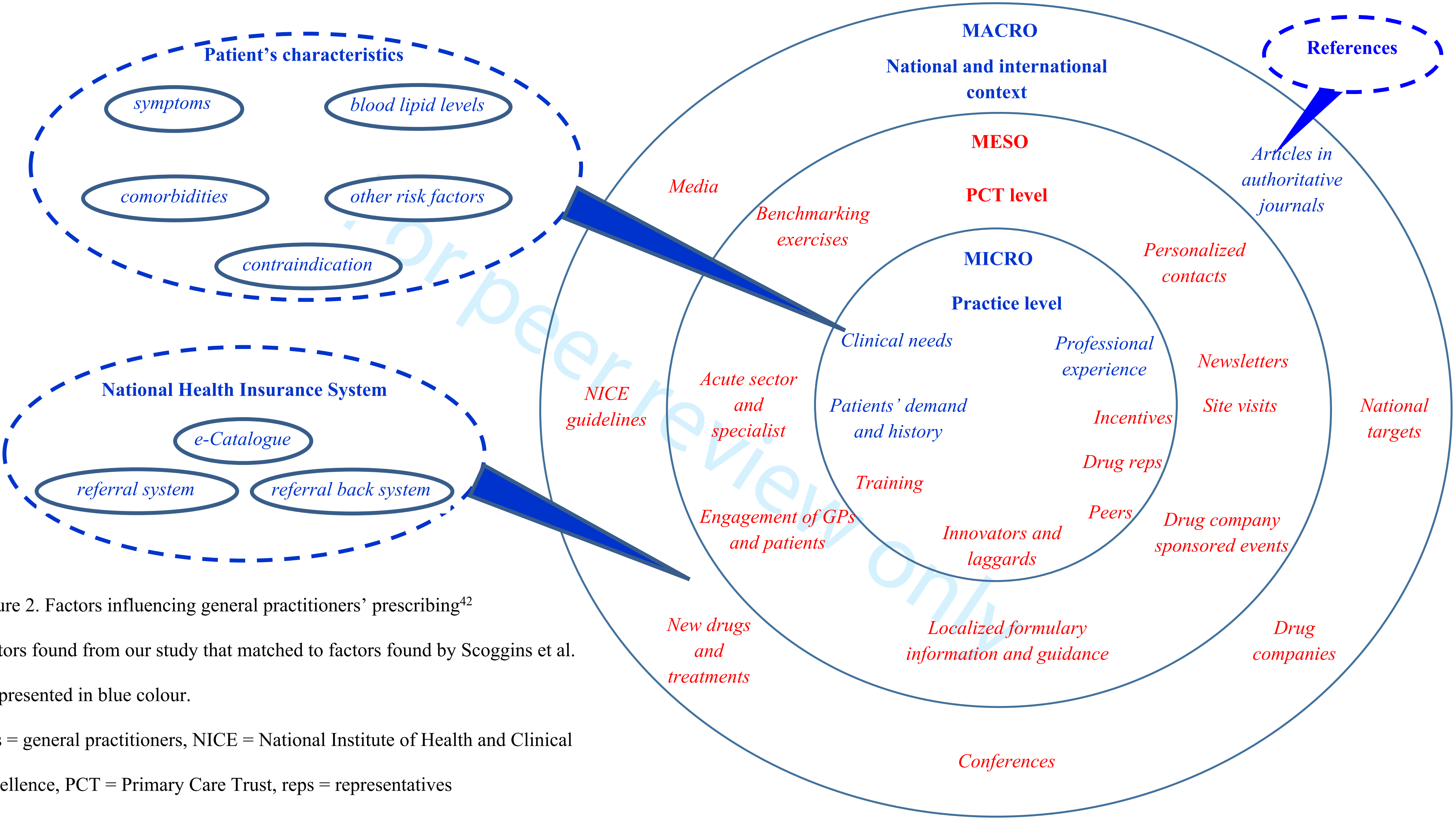

Figure 2. Factors influencing general practitioners' prescribing[42]

Factors found from our study that matched to factors found by Scoggins et al. are presented in blue colour.

GPs = general practitioners, NICE = National Institute of Health and Clinical Excellence, PCT = Primary Care Trust, reps = representatives

For peer review only - http://bmjopen.bmj.com/site/about/guidelines.xhtml

Reporting checklist for qualitative study.

Based on the SRQR guidelines.

Instructions to authors

Complete this checklist by entering the page numbers from your manuscript where readers will find each of the items listed below.

Your article may not currently address all the items on the checklist. Please modify your text to include the missing information. If you are certain that an item does not apply, please write "n/a" and provide a short explanation.

Upload your completed checklist as an extra file when you submit to a journal.

In your methods section, say that you used the SRQRreporting guidelines, and cite them as:

O'Brien BC, Harris IB, Beckman TJ, Reed DA, Cook DA. Standards for reporting qualitative research: a synthesis of recommendations. Acad Med. 2014;89(9):1245-1251.

		Reporting Item	Page Number
**Title**			
	#1	Concise description of the nature and topic of the study identifying the study as qualitative or indicating the approach (e.g. ethnography, grounded theory) or data collection methods (e.g. interview, focus group) is recommended	1

For peer review only

Abstract

	#2	Summary of the key elements of the study using the abstract format of the intended publication; typically includes background, purpose, methods, results and conclusions	2

Introduction

Problem formulation	#3	Description and signifcance of the problem / phenomenon studied: review of relevant theory and empirical work; problem statement	4,5
Purpose or research question	#4	Purpose of the study and specific objectives or questions	5

Methods

Qualitative approach and research paradigm	#5	Qualitative approach (e.g. ethnography, grounded theory, case study, phenomenolgy, narrative research) and guiding theory if appropriate; identifying the research paradigm (e.g. postpositivist, constructivist / interpretivist) is also recommended; rationale. The rationale should briefly discuss the justification for choosing that theory, approach, method or technique rather than other options available; the assumptions and limitations implicit in those choices and how those choices influence study conclusions and transferability.	6

For peer review only

		As appropriate the rationale for several items might be discussed together.	
Researcher characteristics and reflexivity	#6	Researchers' characteristics that may influence the research, including personal attributes, qualifications / experience, relationship with participants, assumptions and / or presuppositions; potential or actual interaction between researchers' characteristics and the research questions, approach, methods, results and / or transferability	6
Context	#7	Setting / site and salient contextual factors; rationale	7
Sampling strategy	#8	How and why research participants, documents, or events were selected; criteria for deciding when no further sampling was necessary (e.g. sampling saturation); rationale	7
Ethical issues pertaining to human subjects	#9	Documentation of approval by an appropriate ethics review board and participant consent, or explanation for lack thereof; other confidentiality and data security issues	5
Data collection methods	#10	Types of data collected; details of data collection procedures including (as appropriate) start and stop dates of data collection and analysis, iterative process, triangulation of sources / methods, and modification of procedures in response to evolving study findings; rationale	7

For peer review only

Data collection instruments and technologies	#11	Description of instruments (e.g. interview guides, questionnaires) and devices (e.g. audio recorders) used for data collection; if / how the instruments(s) changed over the course of the study	7,8,21
Units of study	#12	Number and relevant characteristics of participants, documents, or events included in the study; level of participation (could be reported in results)	8
Data processing	#13	Methods for processing data prior to and during analysis, including transcription, data entry, data management and security, verification of data integrity, data coding, and anonymisation / deidentification of excerpts	8
Data analysis	#14	Process by which inferences, themes, etc. were identified and developed, including the researchers involved in data analysis; usually references a specific paradigm or approach; rationale	6-8
Techniques to enhance trustworthiness	#15	Techniques to enhance trustworthiness and credibility of data analysis (e.g. member checking, audit trail, triangulation); rationale	8
**Results/findings**			
Syntheses and interpretation	#16	Main findings (e.g. interpretations, inferences, and themes); might include development of a theory or model, or integration with prior research or theory	8-12, 24

For peer review only

Links to empirical data	#17	Evidence (e.g. quotes, field notes, text excerpts, photographs) to substantiate analytic findings	8-12
**Discussion**			
Intergration with prior work, implications, transferability and contribution(s) to the field	#18	Short summary of main findings; explanation of how findings and conclusions connect to, support, elaborate on, or challenge conclusions of earlier scholarship; discussion of scope of application / generalizability; identification of unique contributions(s) to scholarship in a discipline or field	13-15
Limitations	#19	Trustworthiness and limitations of findings	13
**Other**			
Conflicts of interest	#20	Potential sources of influence of perceived influence on study conduct and conclusions; how these were managed	16
Funding	#21	Sources of funding and other support; role of funders in data collection, interpretation and reporting	16

The SRQR checklist is distributed with permission of Wolters Kluwer © 2014 by the Association of American Medical Colleges. This checklist was completed on 17. October 2019 using https://www.goodreports.org/, a tool made by the EQUATOR Network in collaboration with Penelope.ai

For peer review only

BMJ Open

Key factors influencing the prescribing of statins: a qualitative study among physicians working in primary health care facilities in Indonesia

Journal:	*BMJ Open*
Manuscript ID	bmjopen-2019-035098.R1
Article Type:	Original research
Date Submitted by the Author:	03-Feb-2020
Complete List of Authors:	Irawati, Sylvi; University of Groningen,<br>Prayudeni, Sari; Universitas Surabaya Kampus UBAYA Tenggilis<br>Rachmawati, Riris; Optima Pharmacy<br>Wita, I ; Udayana University Faculty of Medicine<br>Willfert, Bob; University of Groningen<br>Hak, Eelko; Rijksuniversiteit Groningen, Faculty of Mathematics and Natural Sciences<br>Taxis, Katja; University of Groningen, Faculty of Mathematics and Natural Sciences
**Primary Subject Heading**:	Qualitative research
Secondary Subject Heading:	Cardiovascular medicine, Health policy
Keywords:	Ischaemic heart disease < CARDIOLOGY, Health policy < HEALTH SERVICES ADMINISTRATION & MANAGEMENT, Protocols & guidelines < HEALTH SERVICES ADMINISTRATION & MANAGEMENT, Quality in health care < HEALTH SERVICES ADMINISTRATION & MANAGEMENT, QUALITATIVE RESEARCH

SCHOLARONE™
Manuscripts

I, the Submitting Author has the right to grant and does grant on behalf of all authors of the Work (as defined in the below author licence), an exclusive licence and/or a non-exclusive licence for contributions from authors who are: i) UK Crown employees; ii) where BMJ has agreed a CC-BY licence shall apply, and/or iii) in accordance with the terms applicable for US Federal Government officers or employees acting as part of their official duties; on a worldwide, perpetual, irrevocable, royalty-free basis to BMJ Publishing Group Ltd ("BMJ") its licensees and where the relevant Journal is co-owned by BMJ to the co-owners of the Journal, to publish the Work in this journal and any other BMJ products and to exploit all rights, as set out in our licence.

The Submitting Author accepts and understands that any supply made under these terms is made by BMJ to the Submitting Author unless you are acting as an employee on behalf of your employer or a postgraduate student of an affiliated institution which is paying any applicable article publishing charge ("APC") for Open Access articles. Where the Submitting Author wishes to make the Work available on an Open Access basis (and intends to pay the relevant APC), the terms of reuse of such Open Access shall be governed by a Creative Commons licence – details of these licences and which Creative Commons licence will apply to this Work are set out in our licence referred to above.

Other than as permitted in any relevant BMJ Author's Self Archiving Policies, I confirm this Work has not been accepted for publication elsewhere, is not being considered for publication elsewhere and does not duplicate material already published. I confirm all authors consent to publication of this Work and authorise the granting of this licence.

For peer review only

Key factors influencing the prescribing of statins: a qualitative study among physicians working in primary health care facilities in Indonesia

Sylvi Irawati,[1,2,3*] Sari Prayudeni,[4*] Riris Rachmawati,[5] I Wayan Wita,[6] Bob Willfert,[3,7] Eelko Hak,[3] Katja Taxis[3]

*shared authorship

[1]Universitas Surabaya, Centre for Medicines Information and Pharmaceutical Care, Faculty of Pharmacy, Surabaya, Indonesia

[2]Universitas Surabaya, Department of Clinical and Community Pharmacy, Faculty of Pharmacy, Surabaya, Indonesia

[3]University of Groningen, Groningen Research Institute of Pharmacy, PharmacoTherapy, -Epidemiology & -Economics, Groningen, the Netherlands

[4]Universitas Surabaya, Faculty of Pharmacy, Surabaya, Indonesia

[5]Optima Pharmacy, Surabaya, Indonesia

[6]Udayana University, Department of Cardiovascular Medicine, Denpasar, Indonesia

[7]University of Groningen, Department of Clinical Pharmacy & Pharmacology, University Medical Center Groningen, Groningen, The Netherlands

Corresponding author:

Sylvi Irawati

Email: s.irawati@rug.nl

Phone: +31 638649411

ORCID ID: 0000-0001-6278-9017

For peer review only - http://bmjopen.bmj.com/site/about/guidelines.xhtml

ABSTRACT

Objectives: To elicit key factors influencing physicians' decision to prescribe statins.

Design: A qualitative study using a phenomenological approach within a pragmatism interpretive framework. A combination of purposive and snowball sampling was used to recruit physicians. Data were collected through face-to-face, semi-structured interviews with physicians working in primary health care facilities in a capital of a province in Indonesia. We recorded and verbatim transcribed the interviews. Coding was done independently by two researchers and data were analyzed using phenomenological data analyses. Key factors influencing physicians' decision to prescribe statins were classified into factors at the micro, meso, and macro levels according to the structural model by Scoggins et al.

Participants and setting: Physicians working in primary health care facilities in a capital of a province in Indonesia.

Results: Ten physicians were included in the study. Key factors at the micro level were that physicians knew guidelines in general, but there was uncertainty how to take into account the level of total cholesterol in combination with other cardiovascular risk factors such as diabetes and hypertension. At the macro level, the new National Health Insurance system (NHIS) appeared to facilitate the prescription of statins though more clinical information should be integrated in the system's platform to support appropriate prescribing.

Conclusions: The findings indicate lack of awareness of specific details in current guideline recommendations. Appropriate prescribing of statins should be enhanced using the new NHIS.

Keywords: cardiovascular diseases, physicians, drug prescribing, statins, qualitative research

For peer review only - http://bmjopen.bmj.com/site/about/guidelines.xhtml

Strengths and limitations of this study

- This is the first study to gain insights from physicians on key factors influencing their decision in prescribing statins in a LMIC setting.
- Face-to-face interviews by an interviewer who was known to physicians made it possible to gain more trustworthy information.
- Follow-up interviews, member checking process, independent coders, discussion with several researchers to reach consensus and the use of a theoretical framework improved the credibility of the results.
- Despite code saturation was appropriately reached with a small sample size, a larger sample size is needed to obtain meaning saturation to provide a deeper understanding of the investigated phenomenon.

For peer review only

INTRODUCTION

The use of statins has been endorsed by local and various international guidelines[1–6] to prevent cardiovascular disease (CVD)-related events or death in individuals without history of previous CVD (primary prevention) or with established CVD (secondary prevention).[7–9] But many studies demonstrate underprescribing, and in some cases overprescribing of this drug class. Underprescribing of statins is especially an issue in primary prevention, for example in the groups with moderate-to-very high risk of CVD in the European general population and in an outpatient setting in the United States.[10–13] One small study (N = 243 cases) with medical records data taken from three private hospitals in Jakarta, the capital city of the Republic of Indonesia, a lower-middle-income country (LMIC), suggested patients might not be properly treated with statins. But under- or overprescribing was not clearly defined, because data required to determine the appropriateness to prescribe statins are not routinely measured or well documented.[14 15] Another study in ambulatory diabetes patients with established CVD in Surabaya revealed the phenomenon of underprescribing with 43% of eligible patients not receiving statins.[16] Studies investigating the consequences of under- and overprescribing of statins are scarce. Nevertheless, one case study in a United Kingdom population with a ≥ 20% of 10-y risk of developing CVD, indicating the use of statins for primary prevention of CVD, illustrates that underprescribing will substantially reduce quality-adjusted life years (QALYs) gained over a 9 year period (2006-2014) and overprescribing will certainly increase the cost burden of prescription between £80 million and £117 million annually without certainty whether the benefit of prevented CVD events outweighs the adverse drug reaction.[17]

Factors influencing physicians' prescribing behavior, regardless of the medication being prescribed, has been investigated since the 1950s. Physicians' personal and professional characteristics, pharmaceutical marketing strategies (direct-to-physician or direct-to-consumer marketing), drug information sources, drug characteristics, drug price, and patients' characteristics have been identified as factors influencing physicians' prescribing behavior, although there is inconsistency over the relative importance of each factor.[18–24] More importantly, the majority of studies in this field have been conducted in high-income Western countries with a different culture, health care and health insurance systems compared to LMICs.

In order to reach universal health coverage (UHC) in 2019, the Government of Indonesia launched a new mandatory National Health Insurance System (NHIS) program (*Jaminan Kesehatan*

For peer review only - http://bmjopen.bmj.com/site/about/guidelines.xhtml

Nasional-Kartu Indonesia Sehat, *JKN-KIS*) run by the Indonesian Social Security Agency for Health (*Badan Pengelola Jaminan Sosial*, *BPJS Kesehatan*) in 2014.[25–27] The system facilitates more patients to go to the primary health care facilities first instead of directly visiting specialists in the secondary facilities.[25 28] Moreover, more medicines, listed in the 2014 revised edition of the National Formulary, are insured by the government to improve access to medications for patients.[25 29 30] Based on the National Formulary, two types of statins are insured (pravastatin and simvastatin). An estimation of the total cardiovascular (CV) risk score, which is necessary to guide statin prescribing for primary prevention of CVD, is not mentioned.[29 30] This policy is different compared to the new clinical guideline for dyslipidemic patients issued by the Indonesian Heart Association (IHA) which adapted guidelines issued by the European Society of Cardiology (ESC) and the European Atherosclerosis Society (EAS).[2 31] In these guidelines, statins should be prescribed according to both the level of LDL-C and the total CV risk score, especially in patients with unknown or no history of CVD. Thus, this situation may create confusion for physicians in prescribing statins and increase the risk of under- or overprescribing of statins. In the current qualitative study, we aimed to understand the key factors influencing physicians to prescribe statins, especially in the primary health care facilities in a LMIC.

ETHICS APPROVAL

This study was approved by the local Indonesian Medical Association (IMA). The aim of the study was known to all participants before the interview was started. A written informed consents were obtained from each participants. Participant's personal identities were kept anonymous and all transcripts were stored and maintained confidential.

For peer review only - http://bmjopen.bmj.com/site/about/guidelines.xhtml

METHODS

Study design and framework

This was a qualitative study using a phenomenological approach within a pragmatism interpretive framework. The pragmatism framework focuses on the phenomenon being investigated (key factors influencing physicians in prescribing statins) and the question inquired regarding this phenomenon. This framework allowed choosing the method that worked best in the field to answer the research question.[32] We used the Standards for Reporting Qualitative Research (SRQR) checklist when writing our report.[33]

Study context and setting

As briefly introduced earlier, the Government of Indonesia set a new NHIS conducted by *BPJS Kesehatan*, namely *JKN-KIS*, in 2014. In this system, Indonesia's Ministry of Health also enforced the already existing platforms such as the National Formulary and the e-Catalogue to support cost-effective medicine and medical supply procurement throughout the country. The National Formulary lists medicines reimbursed by BPJS. The e-Catalogue is a national electronic platform required to be used by healthcare facilities for procuring medicines. The e-Catalogue lists medicines with their prices and selected pharmaceutical companies as supplier. Unless the medicines which are needed are not listed in the e-Catalogue, it is mandatory for all health care facilities to purchase medicines from this platform.[25 26]

Indonesia's primary health care is delivered by public and private primary health care facilities. Public primary health care facilities consist of primary health centers (PHCs or *puskesmas*) and their supporting networks (*pustu*, *puskel*, *polindes*, *poskesdes*) owned and organized by District or City Health Offices. Private primary health care facilities consist of individual practice general practitioners (GPs), midwives, and nurses; and private clinics. As a part of new NHIS program in 2014, the national referral system to access these facilities was also established. Figure 1 presents an overall picture of the health care delivery system in Indonesia.[34]

Participants

We viewed the topic of our study was sensitive in nature.[35 36] Prescribing activity in Indonesia is an authority of registered physicians governed by regulations[37 38] and ethical codes.[39 40] There might be issues which would not be frankly expressed due to its sensitivity. Thus, we selected a city, a capital of a

For peer review only - http://bmjopen.bmj.com/site/about/guidelines.xhtml

province in Indonesia, where our study had been welcomed and approved by the local physician associations of the city. We gathered information from physicians working at primary care health facilities in this city. The province was quite small with an area around 6000 ha. The city is the most populous city in the province with around 900,000 inhabitants in 2017, about 21.5% of the province's population. Indonesian regulation allows physicians to conduct their practice at a maximum of three health care facilities (public or private, primary or secondary level).[38] The views of the physicians included in our study reflect their practice to care for patients with and without CVD with possible indications for primary and secondary prevention of CVD.

We obtained the list of physicians' names registered as member of the physician associations of the city, but up-to-date physicians' personal contact details were not available. Therefore, we purposively selected physicians who worked at the primary health care facilities in the capital city who were known to a member of the research team (SP) in a personal (friendship) or professional (acquaintance) manner, and consented to participate in the study. Participating physicians were then asked for contact details of potential other participants (snowball sampling).

Data collection

Baseline characteristics of the participants were collected by interview (age, practice experience, educational period). Initially, an interview guide was developed by SI and SP based on general key factors in prescribing found in the literature (see Table 1).[18] A semi-structured face-to-face interview was conducted with each participant by SP between July 2015 and January 2016. To obtain the key factors considered by physicians in prescribing statins, SP only asked the main question at first. The probing questions were asked after physicians answered the first question in an explanatory way. All interviews were audio-recorded using a tape recorder. The interviewer also took some field notes. The result from the initial interview of each participant was pre-analysed directly by SI and SP to confirm whether it had covered the aim of the study and to determine if a follow-up interview with the same participant was needed. Data collection was discontinued when code saturation was reached as described by Hennink et al.[41]

For peer review only - http://bmjopen.bmj.com/site/about/guidelines.xhtml

Data analysis

The recorded interviews were listened to repeatedly and transcribed verbatim whenever possible. The field notes were checked to add information to the transcripts. To enhance trustworthiness and credibility of data analysis, a technique known as member checking was applied by returning each transcript to the respective participant to confirm the content of the interview.

Using the phenomenological approach, we highlighted and coded keywords which indicated key factors in prescribing statins from the transcripts. The same code was assigned to the same keyword. We categorized the codes in minor subthemes, subthemes and final major themes. The encoding process until obtaining subthemes was conducted independently by SP and RR. Disagreement regarding the codes and subthemes was discussed between SI and SP to reach a consensus. After subthemes were drawn, SP, SI, and IWW discussed major themes and compared the themes with the findings from similar qualitative studies.

The final themes were refined by SI and KT and were mapped into a model that had previously been developed by Scoggins et al.[42] This was a qualitative study, investigating factors influencing GPs to prescribe statins in England using in-depth interviews and focus group discussions. Interviews were done with senior managers primary care service organisations (Primary Care Trusts, PCTs), focus group discussions were done with GPs of those organisations. The factors were classified into macro, meso and micro level (Figure 2).

Patient and Public Involvement

There was no patient and public involvement in the design, conduct, or reporting of our research.

RESULTS

Ten physicians (nine females, one male) were willing to participate in this study. The mean age of all physicians was 31.7 years old (ranged 24 to 56). Five physicians graduated between 2010 and 2015, four between 2000-2010, and one between 1980 and 1990. The mean duration of medical education was 6.6 years (ranged 5 to 12 years). All physicians were GPs. One was in the process of becoming a specialist in internal medicine. All physicians were working at more than one health care facility, four of them practiced at three different health care facilities including at the secondary level. On average, the

For peer review only - http://bmjopen.bmj.com/site/about/guidelines.xhtml

physicians examined around 99 patients per week (range 30 to 250), the mean number of patients with CVD risk was 26.0 patients weekly (range 5 to 100).

Table 2 presents all major themes, subthemes, and minor subthemes derived from transcript analysis. In general, there were two major themes drawn from the analysis: factors operating at (a) micro and (b) macro levels. We did not identify any themes on the meso level. Figure 2 shows the themes mapped into the model by Scoggins et al.[42]

Theme 1: Key factors in statin prescribing at the micro level

The physicians described the key factor influencing their decision to prescribe statins as their inherent understanding of the characteristics of patients which indicate the patient's need for a statin or a contra-indication. There was some uncertainty around these characteristics which was inseparable from other factors like motivation and education. See for further explanation below.

Subtheme: Patient's characteristics

Physicians described several patient's characteristics which they considered to be important for prescribing statins. The most important characteristic was a high level of total cholesterol (TC), other characteristics were clinical symptoms, comorbidities, and other risk factors.

> "From the level of cholesterol of the patient him/herself, if for example, it is more than 200 [mg/dL], for example 210, but it looks like it still can be [controlled] with dietary pattern, I have not prescribed it yet. However, if, for example, there have been symptoms such as [on the] neck and maybe he/she has high blood pressure, usually a medication [is prescribed] – simvastatin 1 x 10 mg. Actually, from my personal perspective, I do not look at the high blood pressure only. Only from the level of cholesterol, if, for example, it has been already high such as up to 250, 260, I give [the medication] even though the blood pressure is not high." (Physician 1)

Interestingly, there is disagreement over the cut-off of the TC level to initiate statins. Other disagreements were also found on how these factors related to each other when influencing the decision to prescribe statins. These variations of considerations were captured in the following quotes:

For peer review only - http://bmjopen.bmj.com/site/about/guidelines.xhtml

"It depends on the patient whether there is a comorbidity or not. If there is a comorbidity, usually [with the level of cholesterol] above 200, [statin] has already been prescribed. If not, maybe above 240. If there is hypertension, diabetes, above 200 also [it will] be directly given." (Physician 8)

"If, for example, the tryglycerides level is above 150, it can be prescribed. Then, after that... tryglycerides and total cholesterol. [For total cholesterol] usually above 200, it is prescribed." (Physician 10)

In prescribing statins, a physician also considered a situation where statin administration was contraindicated as quoted below:

"First, there has to be a clear or an appropriate indication. Then, whether there is a contraindication for the patient to use the medication for example in patients with liver disorder, then in patients who are pregnant – this is also a relative contraindication for statin administration." (Physician 6)

Subtheme: Professional experience of the prescribing physicians

This subtheme focused more on the personal choice of physicians. A mindset of cardiovascular event prevention motivated physicians to prescribe statins as described below:

"I give statins to the patient with high cholesterol lipid not just to decrease the level of the cholesterol. I mean that, you know that patient with high cholesterol lipid has much more risk for cardiovascular incident, because, in my knowledge, if the cholesterol level in the blood is so high, [it] can cause the plaque in the vascular, in the blood vessels. If the plaque becomes unstable, [it] can cause a rupture and make a mismatched perfusion and demand. So, I give the statin for the cholesterolemias, uh... the high cholesterol level in the laboratory test, other reason

For peer review only - http://bmjopen.bmj.com/site/about/guidelines.xhtml

– no other reason, I think. It's the main reason. It's my first reason to give [is] to prevent. It prevents the cardio, the risk of cardiovascular incident." (Physician 4)

"... for statin administration as a medication to manage dyslipidemia, especially in patients with dyslipidemia or in patients with high risk of cardiovascular disease events that it can be as preventive therapy in cardiovascular disease, such as in patients who have already had a coronary heart disease or in patients with diabetes mellitus and… and with a risk, with peripheral artery disease so to say, hence there is a place to give [statin] or in patients with stroke." (Physician 6)

Theme 2: Key factors in statin prescribing operating at the macro level

We found that international information or references framed the physician's mindset of patients who were candidates to receive statins. Furthermore, the NHIS supported or hindered the decision to prescribe statins.

Subtheme: References

References including journals and guidelines were information sources which may frame physicians' knowledge on which patients to prescribe statins for. These factors were not mentioned initially by participants but elicited through probing questions. The sources mentioned included therapeutic guidelines covered during medical education as well as local and global standards.

"I do… as in the standard operating procedure, i.e. giving a healthy lifestyle first… a healthy dietary lifestyle. The standard operating procedure is from sources for example from journals… yes…also with medical references in the past… got from the college and when I was in the hospital [during internship as a junior physician]" (Physician 2)

"In the past Framingham score was used to determine the risk for cardiovascular events. Now there is a new published guideline where [the risk] was not calculated from the Framingham score but [also] saw other risk factors. If not mistaken, [the guideline] was issued by American Heart Association also." (Physician 6)

For peer review only - http://bmjopen.bmj.com/site/about/guidelines.xhtml

> "There is a standard for that. If, for example, [the level of cholesterol] is above 250, the medication is just started. If it is between 200-250, counseling related to dietary pattern and lifestyle only." (Physician 9)

Subtheme: National Health Insurance System

Although the new NHIS means more patients get free access to medicines, there are still criteria to prescribe statins for reimbursement in the National Formulary which may or may not facilitate prescribing. For example, in the National Formulary it was stated that pravastatin was to be given only to hyperlipidemic patients who had LDL-c level > 160 mg/dL, to patients with coronary heart disease, and patients with diabetes and macroalbuminuria.[29 30] This suggested that physicians would tend to prescribe to this type of patients or would prescribe it anyway if the patient did not meet this strict criteria but was considered to need a statin with the consequence it would not be reimbursed by BPJS.

> "The price is cheap, [it] is easy to obtain... in terms of it is available everywhere, then also available in generic. This also does not make it difficult for some patients even more when retired elderly patients do not have money but using *BPJS*. So, I think that is my consideration." (Physician 5)

> "*BPJS* will only reimburse the administration of statins for LDL above 160. Therefore, if it is under 160, patients usually buy it by themselves, so we prescribe. Meanwhile, specialists in hospitals usually prescribe but this is not reimbursed by BPJS... so we help by prescribe it again." (Physician 8)

> "No, if indeed only the cholesterol is high, I think no need to refer [the patient]. No diabetes, nothing... we usually prescribe directly, unless when simvastatin is incidentally not available in *Puskesmas*... he/she [the patient] insists because he/she has a right... sometimes the medication is not available. He/she has the right... insurance right, for example he/she wants a referral, we

For peer review only - http://bmjopen.bmj.com/site/about/guidelines.xhtml

refer. However, there he/she also does not certainly obtain [a statin] if the LDL is under 160." (Physician 8)

A theme categorized under Pandora Box

We attempted to elicit the impact of pharmaceutical companies and their marketing strategy related to statin prescribing, but physicians preferred to not discuss this topic with the researcher.

DISCUSSION

Physicians' decision to prescribe statins was influenced by factors operating at the micro and macro levels which mapped well on the framework developed by Scoggins et al (2006) in a study on the factors influencing GPs prescribing of statins in primary care in England. The key micro level factor was a set of patient's characteristics, especially the high level of TC. At the macro level, physicians explained that their knowledge on prescribing was grounded in their education at university as well as reading guidelines or other reference sources such as medical journals. Another macro level factor was the new NHIS which facilitated or limited the prescribing of statins.

The strength of this study lies in the use of a qualitative method to explore processes behind the behavior of prescribing statins in Indonesia, a LMIC in transition to achieve UHC. The validity of the data were improved by follow-up interviews, member checking process, independent coders, and discussion with several researchers to reach consensus. Although we have reached code saturation with our interviews, we only interviewed a relatively small number of physicians having relatively homogenous characteristics from one city in one province in Indonesia. Furthermore, the interviewer knew a number of the physicians personally and this may have had an impact on the willingness to share sensitive information. The interviewer had the subjective impression that those interviews were longer and contained more information than the interviews with the physicians which she met the first time. Owton and Allen-Collinson discussed the use of friendship approach for data collection in ethnographic research. Although they noted interactional challenges during interviews, e.g. emotional overload and power struggle, due to a role conflict being a friend and a researcher, but overall, this approach revealed more personal and sensitive information in some encounters.[43] Nevertheless, several of our findings are comparable with other qualitative studies. Therefore, although the current study is explorative, our

For peer review only - http://bmjopen.bmj.com/site/about/guidelines.xhtml

findings are the foundation for a future quantitative study, e.g. surveys and interventions, in Indonesia. More qualitative work on eliciting factors influencing physician's prescribing behavior in other Asian regions and other therapeutic areas could complement our findings.

Similar to Scoggins et al. (2006), our study found the clinical need of patients as an important factor to consider prescribing statins. However, physicians in our study described the clinical needs in more detail, mainly the level of TC and other risk factors potentially increasing the risk of CVD. Of note, the physicians did not specifically differentiate between statin prescribed for primary or secondary prevention of CVD. Interestingly, the range of cholesterol levels to start statins ranged from 200 mg/dL to 260 mg/dL and there was also a variation in taking other CVD risk factors into account. For example, one physician decided to give a statin when the TC level ≥ 210 mg/dL and the patient also had clinical symptoms and comorbidities while another physician only prescribed statins when the patient's TC level was ≥ 260 mg/dL without the presence of comorbidities. This practice was not in line with the local guideline to initiate statin therapy.[2] The patient's total CV risk calculation was only acknowledged once after probing. This variation may lead to under- or overprescribing of statins. The hesitation and inconsistency in using risk assessment tools as a guidance to initiate statin therapy has also been revealed in another qualitative study performed among GPs in the United Kingdom (UK) which showed that GPs found it difficult to interpret the tool for Asian patients and therefore preferred to assess the risk intuitively.[44] The physicians in our study had some general background knowledge on statin prescribing which was by and large in line with the National Formulary. They did not mention the use of the national guideline issued by the Indonesian Heart Association as sources to guide statin prescribing. This raises the question whether this guideline is sufficiently promoted.

Our general findings are also in agreement with a report from the World Health Organization (WHO) on the drug use situation in Indonesia in 2011. It reported non-uniform sources of drug information used by physicians; they could be old text books from medical school or the internet with little use of up-to-date sources of independent drug information. The national standard treatment guideline for PHCs was not used much by physicians.[45] Even if it was used, there was no recommendation to use statins in patients with coronary heart disease as the guideline was developed when statins had not routinely been used in clinical practice.[46]

For peer review only - http://bmjopen.bmj.com/site/about/guidelines.xhtml

We could not disclose the role of medical representatives or pharmaceutical companies as shown in other studies [24 42] in influencing statin prescribing. Most likely the interviewees perceived this topic as too controversial within both the medical profession and broader community to be willing to discuss it.[47] Nevertheless, the WHO report suggested medical representatives from pharmaceutical companies as one of probable sources of drug information used by most physicians.[45] As the WHO report is based on a situational analysis conducted in 2011 where the NHIS was still not integrated, it is not possible to know to what extent the role of pharmaceutical companies has changed. As this question clearly created an ethical dilemma, therefore, a collaboration with the Ministry of Health and professional bodies and councils is needed to provide a less biased answer.

Patients' demand or request for medicines is a factor influencing physicians' prescribing behavior according to Scoggins et al. (2006). This factor was not mentioned in our interviews although there was an indication that patient's insistence may influence the physician to meet the patient's expectation, e.g. in granting a referral. The lack of involvement of the patients' voice in the physicians' decision can be caused by differences in physician-patient communication style which may be rooted in cultural differences between Southeast Asians and Westerners.[48 49] The majority of studies exploring factors influencing physicians' prescribing behavior come from Western countries where the physician-patient communication style is often based on a partnership style with the concept of equity.[20 24 42 44 50 51] Southeast Asian physicians commonly tend to communicate in a paternalistic or one-way style, which gives the physician a more dominant role towards the patient. The culture of Southeast Asian physicians in keeping a social distance supports this communication style.[48 49]

We found that the NHIS had a considerable influence on physicians' prescribing decisions, in particular on the choice of generics. This is in line with the concept from Scoggins et al. (2006) that prescribing decisions are influenced by factors operating at national context or macro level. However, Scoggins et al. (2006) only list national and international evidence based on authoritative journals, national guidelines, national media, and the development of new treatments as factors. This difference may be explained because of differences in organizing health care and reimbursement of medication between Indonesian and England.

In contrast to Scoggins et al.,[42] we did not identify any factors at the meso level such as benchmarking between different primary care practices, collaborations between primary care and

For peer review only - http://bmjopen.bmj.com/site/about/guidelines.xhtml

specialists and regional formularies. This is understandable as strengthening the primary care sector is a recently developed strategy with the new NHIS in Indonesia. Therefore, activities which are common practice in the English primary care system at the meso level, have not been established in Indonesia yet. This finding is an opportunity for the government to work on the meso level to strengthen rational prescribing behaviour.

The findings from our study indicate that physicians have a lack of awareness of specific guideline recommendations, especially on the use of LDL-C level and total CV risk calculation as decisive factors to prescribe statins. Improved access to statins facilitated by the government through the new NHIS seemed to be a good first step in fostering rational prescribing. A next step would be to include the recommendations from clinical guidelines issued by IHA into this system, especially through the National Formulary.

DATA AVAILABILITY STATEMENT

Data from the interviews that correspond to the results of this study, after de-identification of study participants, can be made available upon request at the beginning 9 months and ending 36 months following manuscript publication for researchers who provide a methodologically sound proposal. Proposal should be directed to the corresponding author of this study (sylviirawati.2010@gmail.com).

ACKNOWLEDGEMENTS

The authors would like to thank to all physicians participated in this study.

CONTRIBUTORS

SI, SP, and IWW conceived and designed the study. SP carried out the interviews. SI, SP, RR, EH and KT analysed and interpreted the data. SI and SP drafted the manuscript. IWW, BW, EH, and KT gave critical inputs to the intellectual content in the drafted manuscript. All authors read, revised, and approved the final manuscript. SP had full access to all of the data in the study. All authors can take responsibility for the integrity of the work.

For peer review only - http://bmjopen.bmj.com/site/about/guidelines.xhtml

FUNDING

This research received no specific grant from any funding agency in the public, commercial, or not-for-profit sectors. SI received support from the Indonesia Endowment Fund for Education (Lembaga Pengelola Dana Pendidikan, LPDP), the Ministry of Finance of Republic of Indonesia, in the form of scholarship for her PhD program. The LPDP had no role in research design, data collection, analysis and interpretation, or manuscript writing and publication.

COMPETING INTERESTS

None declared

REFERENCES

1. World Health Organization. Prevention of cardiovascular disease: guidelines for assessment and management of cardiovascular risk. Geneva: WHO Press; 2007.
2. Erwinanto, Santoso A, Putranto JNE, et al. Pedoman tatalaksana dislipidemia. J Kardiol Indones [Internet]. 2013;34(4):245–70. Available from: http://jki.or.id.
3. JBS3 Board. Joint British Societies' consensus recommendations for the prevention of cardiovascular disease (JBS3). Heart. 2014;100:ii1–67.
4. Piepoli MF, Hoes AW, Agewall S, et al. 2016 European guidelines on cardiovascular disease prevention in clinical practice. Eur Heart J. 2016;37(29):2315–81.
5. Stone NJ, Robinson J, Lichtenstein AH, et al. 2013 ACC/AHA guideline on the treatment of blood cholesterol to reduce atherosclerotic cardiovascular risk in adults: a report of the American College of Cardiology/American Heart Association Task Force on Practice Guidelines. Circulation. 2014;129::S1–S45.
6. Catapano AL, Graham I, De Backer G, et al. 2016 ESC/EAS guidelines for the management of dyslipidaemias. Eur Heart J. 2016;37(39):2999–3058.
7. Chou R, Dana T, Blazina I, et al. Statins for prevention of cardiovascular disease in adults. JAMA. 2016;316(19):2008–24.
8. Mihaylova B, Emberson J, Blackwell L, et al. The effects of lowering LDL cholesterol with statin therapy in people at low risk of vascular disease: meta-analysis of individual data from 27

For peer review only

randomised trials. Lancet. 2012;380(9841):581–90.

9. Taylor F, Huffman M, Macedo A, et al. Statins for the primary prevention of cardiovascular disease. Cochrane Database Syst Rev. 2013;(1):1–97.

10. Tonstad S, Rosvold EO, Furu K, et al. Undertreatment and overtreatment with statins: the Oslo Health Study 2000-2001. J Intern Med. 2004;255(4):494–502.

11. Smith M, Cox E, Bartell J. Overprescribing of lipid lowering agents. Qual Saf Health Care. 2006;15(4):251–7.

12. Verma A, Visintainer P, Elarabi M, et al. Overtreatment and undertreatment of hyperlipidemia in the outpatient setting. South Med J. 2012;105(7):329–33.

13. Langsted A, Freiberg JJ, Nordestgaard BG. Extent of undertreatment and overtreatment with cholesterol-lowering therapy according to European guidelines in 92,348 Danes without ischemic cardiovascular disease and diabetes in 2004–2014. Atherosclerosis. 2017;257:9–15.

14. Simatupang A, Irianto A, Simanjuntak W, et al. Pattern of statin use in several hospital in Jakarta: a cross sectional study. J Kedokt Yars. 2006;14(3):223–9.

15. Third report of the National Cholesterol Education Program (NCEP) expert panel on detection, evaluation, and treatment of high blood cholesterol in adults (Adult Treatment Panel III) final report. Circulation. 2002;106(25):3143–421.

16. Lie PGCE, Irawati S, Presley B. Prevention of cardiovascular disease in diabetes mellitus outpatient: focusing on antiplatelet, statins and irrational antihypertensive drug use. Indones J Clin Pharm. 2016;5(3):169–83.

17. Hodgson R, Biswas M, Morgan P. Costs and consequences of lack of adherence to prescribing guidelines for statins in the UK: a value of implementation analysis (PCV41). Value Heal. 2017;20(5):A266.

18. Groves KE, MacKinnon NJ, Sketris I. Prescribing behavior. In: Rickles N, Albert I, Mickey C, editors. Social and behavioral aspects of pharmaceutical care. 2nd ed. Sudburry, USA: Jones and Bartlett Publishers; 2010. p. 141–76.

19. Hemminki E. Factors influencing drug prescribing: inquiry into research strategy. Drug Intell Clin Pharm. 1976;10(6):321–9.

20. Hemminki E. Review of literature on the factors affecting drug prescribing. Soc Sci Med.

For peer review only

For peer review only - http://bmjopen.bmj.com/site/about/guidelines.xhtml

1975;9(2):111–5.

21. Theodorou M, Tsiantou V, Pavlakis A, et al. Factors influencing prescribing behaviour of physicians in Greece and Cyprus: results from a questionnaire based survey. BMC Health Serv Res. 2009;9:150. doi: 10.1186/1472-6963-9-150.

22. Christensen DB, Bush PJ. Drug prescribing: patterns, problems and proposals. Soc Sci Med. 1981;15(3 Pt 2):343–55.

23. Schumock GT, Walton SM, Park HY, et al. Factors that influence prescribing decisions. Ann Pharmacother. 2004;38(4):557–62.

24. Murshid MA, Mohaidin Z. Models and theories of prescribing decisions: a review and suggested a new model. Pharm Pract (Granada). 2017;15(2):1–12.

25. Agustina R, Dartanto T, Sitompul R, et al. Universal health coverage in Indonesia: concept, progress, and challenges. Lancet. 2019;393(10166):75–102.

26. Wasir R, Irawati S, Makady A, et al. Use of medicine pricing and reimbursement policies for universal health coverage in Indonesia. PLoS One. 2019;14(2):1–19.

27. Wirtz T. The new mandatory health insurance scheme: taking stock one year after the introduction. Insurance Newsletter. PT Ernst & Young Indonesia; 2015.

28. Ekawati FM, Claramita M, Hort K, et al. Patients' experience of using primary care services in the context of Indonesian universal health coverage reforms. Asia Pac Fam Med. 2017;16(1):1–10.

29. Keputusan Menteri Kesehatan Republik Indonesia tentang Formularium Nasional. No. 328/MENKES/SK/VIII/2013. Jakarta; 2013.

30. Keputusan Menteri Kesehatan Republik Indonesia tentang perubahan atas Keputusan Menteri Kesehatan No. 328/MENKES/SK/IX/2013 tentang Formularium Nasional. No. 159/MENKES/SK/V/2014. Jakarta; 2014.

31. Reiner Ž, Catapano AL, De Backer G, et al. ESC/EAS guidelines for the management of dyslipidaemias. Eur Heart J. 2011;32(14):1769–818.

32. Creswell J, Poth CN. Qualitative inquiry and research design: choosing among five approaches. 4th ed. Los Angeles: SAGE Publication, Inc.; 2018.

33. O'Brien BC, Harris IB, Beckman TJ, et al. Standards for reporting qualitative research: a

For peer review only

For peer review only - http://bmjopen.bmj.com/site/about/guidelines.xhtml

synthesis of recommendations. Acad Med. 2014;89(9):1245–51.

34. Claramita M, Syah NA, Ekawati FM, et al. Primary health care systems (Primasys): case study from Indonesia, abridged version. Geneva: World Health Organization; 2017.

35. Budianto A. Peresepan dokter: organized crime? J Huk Pembang. 2009;39(4):511–30.

36. Muh Fadhli W, Anisah S. Legal responsibilities of doctors and pharmacists in the services of recipes. Media Farm. 2016;13(1):61–87.

37. Undang-Undang Republik Indonesia tentang Praktik Kedokteran. No. 29. 2004.

38. Peraturan Menteri Kesehatan Republik Indonesia tentang Izin Praktik dan Pelaksanaan Praktik Kedokteran. No. 2052/MENKES/PER/X/2011. Jakarta; 2011.

39. Purwadianto A, Soetedjo, Gunawan S, et al. Kode etik kedokteran Indonesia. Jakarta: Pengurus Besar Ikatan Dokter Indonesia; 2012.

40. Badan Etik dan Pembelaan Anggota Pengurus Pusat PERKI. Pedoman kode etik dokter Perhimpunan Kardiologi Indonesia (PERKI). 1st ed. Jakarta: PP. PERKI; 2014.

41. Hennink MM, Kaiser BN, Marconi VC. Code saturation versus meaning saturation: how many interviews are enough? Qual Health Res. 2017;27(4):591–608.

42. Scoggins A, Tiessen J, Ling T. Prescribing in primary care: understanding what shapes GPs' prescribing choices and how might these be changed. [Internet]. Cambridge; 2006 [cited 2019 Feb 25]. p. 1–59. Available from: https://www.rand.org/content/dam/rand/pubs/technical_reports/2007/RAND_TR443.pdf

43. Owton H, Allen-Collinson J. Close but not too close: friendship as method(ology) in ethnographic research encounters. J Contemp Ethnogr. 2014;43(3):283–305.

44. Kedward J, Dakin L. A qualitative study of barriers to the use of statins and the implementation of coronary heart disease prevention in primary care. Br J Gen Pract. 2003;53(494):684–9.

45. Holloway K. Pharmaceuticals in health care delivery [Internet]. World Health Organization. 2011 [cited 2019 Sep 30]. Available from: http://searo.who.int/entity/medicines/indonesia_situational_analysis.pdf?ua=1

46. Pedoman pengobatan dasar di Puskesmas 2007. Jakarta: Departemen Kesehatan RI; 2007.

47. Komesaroff PA, Kerridge IH. Ethical issues concerning the relationships between medical practitioners and the pharmaceutical industry. Med J Aust. 2002;176(3):118–21.

For peer review only - http://bmjopen.bmj.com/site/about/guidelines.xhtml

48. Claramita M, van Dalen J, van der Vleuten CP. Doctors in a Southeast Asian country communicate sub-optimally regardless of patients' educational background. Patient Educ Couns [Internet]. 2011;85(3):e169–74. Available from: http://dx.doi.org/10.1016/j.pec.2011.02.002

49. Claramita M, Nugraheni MDF, van Dalen J, et al. Doctor-patient communication in Southeast Asia: a different culture? Adv Heal Sci Educ. 2013;18(1):15–31.

50. Grant A, Sullivan F, Dowell J. An ethnographic exploration of influences on prescribing in general practice: why is there variation in prescribing practices? Implement Sci. 2013;8(1):2013.

51. AB E, Denig P, Van Vliet T, et al. Reasons of general practitioners for not prescribing lipid-lowering medication to patients with diabetes: a qualitative study. BMC Fam Pract. 2009;10:1–7.

52. Andriani H, Liao CY, Kuo HW. Association of maternal and child health center (Posyandu) availability with child weight status in Indonesia: a national study. Int J Environ Res Public Health. 2016;13(3):293. doi: 10.3390/ijerph13030293.

For peer review only

Table 1. Interview guide

Questions
Main question
• Which factors do you consider when prescribing statins to the patient ?
Probing question (not necessarily being asked, depends on answers from the main question)
• Which therapeutic guideline did you usually use in prescribing statins ?
• How do you assess a total cardiovascular risk factor ?
• Which type of statins do you often prescribe ?
• Which statin dose and duration of use you prescribe ?
• How about the relation to pharmaceutical company promotion/advertising ?
• Does the regulation in the practice setting limit the decision of therapy ?
• Have you ever found patients complaining about the cost of statins ?
• Are you having difficulties in arranging time for a consultation with the patient because there are many queues ?
• Does an insurance system such as the new national health insurance program limit you to prescribe statins to patients who need it ?

For peer review only

For peer review only - http://bmjopen.bmj.com/site/about/guidelines.xhtml

Table 2. Themes, subthemes and codings of factors influencing statin prescribing

Major themes	Subthemes	Minor subthemes	Specific keywords
Micro level	Patient's characteristics	Blood lipid levels	Total cholesterol (varied among participants: from 200 to 260 mg/dL; triglycerides), high LDL-c, low HDL-c, a combination of different lipid parameter
		Symptoms	Pain (varied in location: back of the neck, neck, shoulder), stiffness (neck, shoulder), warmth (neck), tingling (palms, feet), dizziness
		Comorbidities	Hypertension, diabetes, metabolic syndrome, heart disease
		Contraindication	Pregnancy, liver disease
		Other risk factors	Other risk factor without specifying the factor
	Professional experience	Primary and secondary prevention of CVD	Prevention, CVD prevention
Macro level	References	Standard	Standard
		Guideline	Guideline from ACC/AHA
		Journal	Article in journal
	National Health Insurance System	BPJS	BPJS
		e-Catalogue	e-Catalogue
		Referral system, referral back system	patient referral

ACC, American College of Cardiology; AHA, American Heart Association; BPJS, Badan Pengelola Jaminan Sosial; CVD, cardiovascular disease; HDL-c, high density lipoprotein cholesterol; LDL-c, low density lipoprotein cholesterol

For peer review only - http://bmjopen.bmj.com/site/about/guidelines.xhtml

Legend Figure 1

Health care delivery system in Indonesia, figure was modified from Claramita et al.[34]

Puskesmas is a public primary health center. *Posyandu* is a service organized by volunteers (*Kader*) to deliver health program for vulnerable groups, especially mother and children, locally. *Posyandu* is supervised by *Puskesmas*.[52]

Legend Figure 2

Factors influencing general practitioners' prescribing.

Factors found from our study that matched to factors found by Scoggins et al.[42] are presented in blue colour.

BPJS = Badan Pengelola Jaminan Sosial, GPs = general practitioners, NICE = National Institute of Health and Clinical Excellence, PCT = Primary Care Trust, reps = representatives.

For peer review only - http://bmjopen.bmj.com/site/about/guidelines.xhtml

For peer review only

For peer review only - http://bmjopen.bmj.com/site/about/guidelines.xhtml

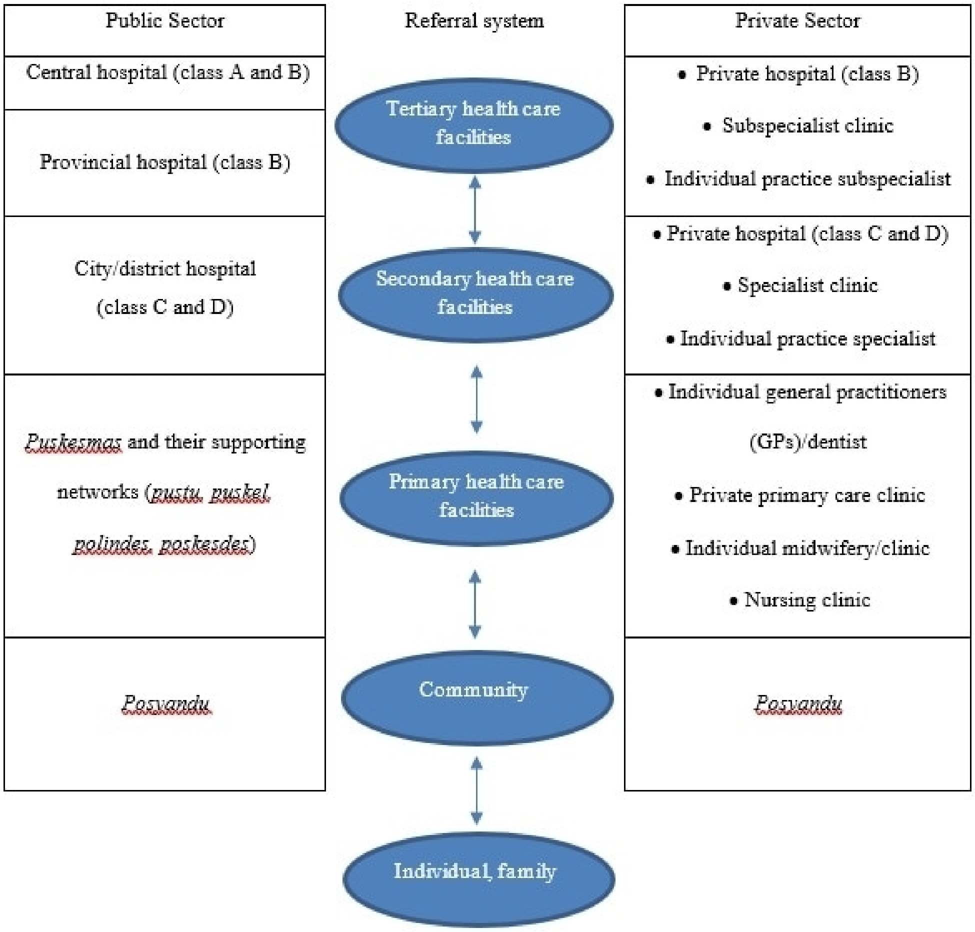

Figure1. Health care delivery system in Indonesia, figure was modified from Claramita et al.[34] Puskesmas is a public primary health center. Posyandu is a service organized by volunteers (Kader) to deliver health program for vulnerable groups, especially mother and children, locally. Posyandu is supervised by Puskesmas.[52]

50x48mm (300 x 300 DPI)

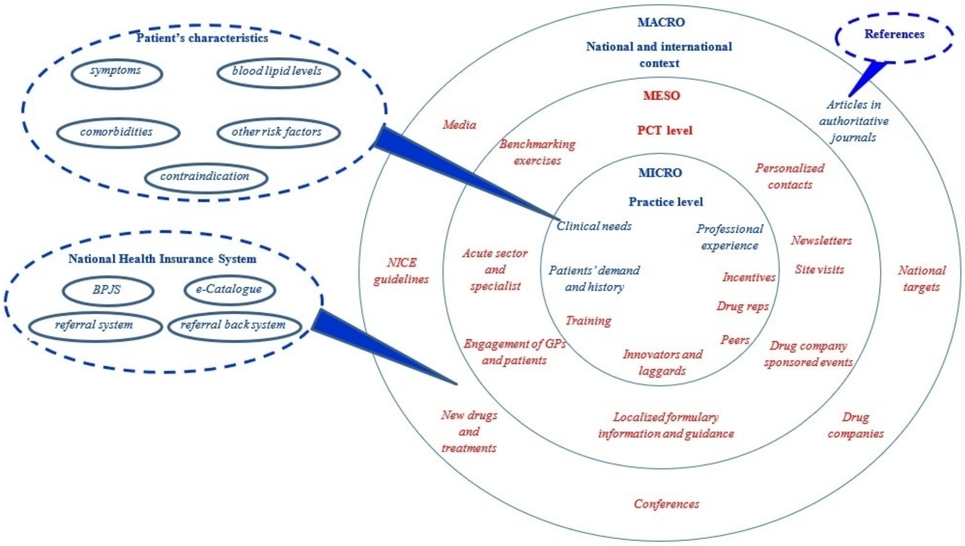

Figure 2. Factors influencing general practitioners' prescribing.
Factors found from our study that matched to factors found by Scoggins et al.[42] are presented in blue colour.
BPJS = Badan Pengelola Jaminan Sosial, GPs = general practitioners, NICE = National Institute of Health and Clinical Excellence, PCT = Primary Care Trust, reps = representatives.

87x48mm (300 x 300 DPI)

Reporting checklist for qualitative study.

Based on the SRQR guidelines.

Instructions to authors

Complete this checklist by entering the page numbers from your manuscript where readers will find each of the items listed below.

Your article may not currently address all the items on the checklist. Please modify your text to include the missing information. If you are certain that an item does not apply, please write "n/a" and provide a short explanation.

Upload your completed checklist as an extra file when you submit to a journal.

In your methods section, say that you used the SRQRreporting guidelines, and cite them as:

O'Brien BC, Harris IB, Beckman TJ, Reed DA, Cook DA. Standards for reporting qualitative research: a synthesis of recommendations. Acad Med. 2014;89(9):1245-1251.

		Reporting Item	Page Number
**Title**			
	#1	Concise description of the nature and topic of the study identifying the study as qualitative or indicating the approach (e.g. ethnography, grounded theory) or data collection methods (e.g. interview, focus group) is recommended	1

For peer review only

**Abstract**			
	#2	Summary of the key elements of the study using the abstract format of the intended publication; typically includes background, purpose, methods, results and conclusions	2
**Introduction**			
Problem formulation	#3	Description and signifcance of the problem / phenomenon studied: review of relevant theory and empirical work; problem statement	4,5
Purpose or research question	#4	Purpose of the study and specific objectives or questions	5
**Methods**			
Qualitative approach and research paradigm	#5	Qualitative approach (e.g. ethnography, grounded theory, case study, phenomenolgy, narrative research) and guiding theory if appropriate; identifying the research paradigm (e.g. postpositivist, constructivist / interpretivist) is also recommended; rationale. The rationale should briefly discuss the justification for choosing that theory, approach, method or technique rather than other options available; the assumptions and limitations implicit in those choices and how those choices influence study conclusions and transferability.	6

		As appropriate the rationale for several items might be discussed together.	
Researcher characteristics and reflexivity	#6	Researchers' characteristics that may influence the research, including personal attributes, qualifications / experience, relationship with participants, assumptions and / or presuppositions; potential or actual interaction between researchers' characteristics and the research questions, approach, methods, results and / or transferability	6
Context	#7	Setting / site and salient contextual factors; rationale	7
Sampling strategy	#8	How and why research participants, documents, or events were selected; criteria for deciding when no further sampling was necessary (e.g. sampling saturation); rationale	7
Ethical issues pertaining to human subjects	#9	Documentation of approval by an appropriate ethics review board and participant consent, or explanation for lack thereof; other confidentiality and data security issues	5
Data collection methods	#10	Types of data collected; details of data collection procedures including (as appropriate) start and stop dates of data collection and analysis, iterative process, triangulation of sources / methods, and modification of procedures in response to evolving study findings; rationale	7

For peer review only

Data collection instruments and technologies	#11	Description of instruments (e.g. interview guides, questionnaires) and devices (e.g. audio recorders) used for data collection; if / how the instruments(s) changed over the course of the study	7,8,21
Units of study	#12	Number and relevant characteristics of participants, documents, or events included in the study; level of participation (could be reported in results)	8
Data processing	#13	Methods for processing data prior to and during analysis, including transcription, data entry, data management and security, verification of data integrity, data coding, and anonymisation / deidentification of excerpts	8
Data analysis	#14	Process by which inferences, themes, etc. were identified and developed, including the researchers involved in data analysis; usually references a specific paradigm or approach; rationale	6-8
Techniques to enhance trustworthiness	#15	Techniques to enhance trustworthiness and credibility of data analysis (e.g. member checking, audit trail, triangulation); rationale	8

Results/findings

Syntheses and interpretation	#16	Main findings (e.g. interpretations, inferences, and themes); might include development of a theory or model, or integration with prior research or theory	8-12, 23, Figure 2

For peer review only

Links to empirical data	#17	Evidence (e.g. quotes, field notes, text excerpts, photographs) to substantiate analytic findings	8-12
**Discussion**			
Intergration with prior work, implications, transferability and contribution(s) to the field	#18	Short summary of main findings; explanation of how findings and conclusions connect to, support, elaborate on, or challenge conclusions of earlier scholarship; discussion of scope of application / generalizability; identification of unique contributions(s) to scholarship in a discipline or field	13-15
Limitations	#19	Trustworthiness and limitations of findings	13
**Other**			
Conflicts of interest	#20	Potential sources of influence of perceived influence on study conduct and conclusions; how these were managed	16
Funding	#21	Sources of funding and other support; role of funders in data collection, interpretation and reporting	16

The SRQR checklist is distributed with permission of Wolters Kluwer © 2014 by the Association of American Medical Colleges. This checklist was completed on 17. October 2019 using https://www.goodreports.org/, a tool made by the EQUATOR Network in collaboration with Penelope.ai

For peer review only
